# A thermosensor FUST1 primes heat-induced stress granule formation via biomolecular condensation in *Arabidopsis*

**DOI:** 10.1038/s41422-025-01125-4

**Published:** 2025-05-14

**Authors:** Pan Geng, Changxuan Li, Xuebo Quan, Jiaxuan Peng, Zhiying Yao, Yunhe Wang, Ming Yang, Yanning Wang, Yunfan Jin, Yan Xiong, Hongtao Liu, Yijun Qi, Peiguo Yang, Kai Huang, Xiaofeng Fang

**Affiliations:** 1 Center for Plant Biology, School of Life Sciences, Tsinghua University, Beijing, China; 2 Institute of Systems and Physical Biology, Shenzhen Bay Laboratory, Shenzhen, Guangdong China; 3 School of Life Sciences, Westlake University, Hangzhou, Zhejiang China; 4 Synthetic Biology Center, Haixia Institute of Science and Technology, Fujian Agriculture and Forestry University, Fuzhou, Fujian China; 5 College of Life Sciences and Oceanography, Shenzhen University, Shenzhen, Guangdong China

**Keywords:** Plant molecular biology, RNA metabolism, Plant signalling

## Abstract

The ability to sense cellular temperature and induce physiological changes is pivotal for plants to cope with warming climate. Biomolecular condensation is emerging as a thermo-sensing mechanism, but the underlying molecular basis remains elusive. Here we show that an intrinsically disordered protein FUST1 senses heat via its condensation in *Arabidopsis thaliana*. Heat-dependent condensation of FUST1 is primarily determined by its prion-like domain (PrLD). All-atom molecular dynamics simulation and experimental validation reveal that PrLD encodes a thermo-switch, experiencing lock-to-open conformational changes that control the intermolecular contacts. FUST1 interacts with integral stress granule (SG) components and localizes in the SGs. Importantly, FUST1 condensation is autonomous and precedes condensation of several known SG markers and is indispensable for SG assembly. Loss of FUST1 significantly delays SG assembly and impairs both basal and acquired heat tolerance. These findings illuminate the molecular basis for thermo-sensing by biomolecular condensation and shed light on the molecular mechanism of heat stress granule assembly.

## Introduction

Heat stress is one of the most challenging environmental risks in the coming decades due to climate change.^1,2^ Unlike mammals, plants are unable to maintain their body temperature at a constant level. Heat alters the structure and function of macromolecules, induces protein misfolding and triggers the production of reactive oxygen species (ROS), which can cause plant cell death.^3^ Therefore, understanding how plants sense and respond to high temperature stress is instrumental to crop engineering or breeding via optimal tilt of the balance between defense and growth.

The sensing of heat by plant cells is widely distributed throughout subcellular compartments and regulatory nodes.^4–7^ Recently, the reversible condensation of proteins is emerging as a crucial mechanism of thermosensing both in plants^8–10^ and yeast.^11–13^ In these cases, the condensation involves weak and multivalent interactions from intrinsically disordered regions (IDRs)^8–10,12,13^ as well as stronger interactions provided by the modular domains.^10–13^ Conformational rearrangements were reported to be a key feature during temperature sensing.^10,11^ Nevertheless, the atomic basis underlying temperature sensing by condensation remains largely elusive.

Stress granules (SGs) are one prominent type of conserved cytoplasmic biomolecular condensates that form in response to a variety of stressors.^14,15^ SGs assemble via liquid‒liquid phase separation (LLPS) driven by dynamic and promiscuous protein‒protein and protein‒RNA interactions.^14^ These interactions are distributed unevenly, with G3BP as the central node.^16^ The current model in mammalian cells holds that G3BP is a molecular switch: stalled translation leads to a rise in cellular free RNA concentrations, which, above a certain threshold, triggers G3BP conformational change that favors G3BP‒RNA interaction. This then results in RNA-dependent LLPS that seeds SG assembly.^16–19^ In plants, the general composition of heat-induced SGs has been determined, but how heat signal is transmitted to SG assembly is yet unknown.

Here, we discover a novel protein, FUST1, that directly senses heat and drives heat-induced SG assembly. We employ all-atom molecular dynamics simulation (DM) in combination with biophysical studies to show that FUST1 contains a thermo-switch that undergoes lock-to-open conformational change upon temperature increase. Imaging analyses reveal that FUST1 condensation in vivo is autonomous, precedes the condensation of several known SG markers and is indispensable for SG assembly. Ablation of FUST1 function significantly delays SG formation. We conclude that FUST1 is a molecular thermosensor that drives heat-induced SG assembly in plants.

## Results

### FUST1 is an uncharacterized protein that undergoes heat-dependent condensation

**Fig. 1:**
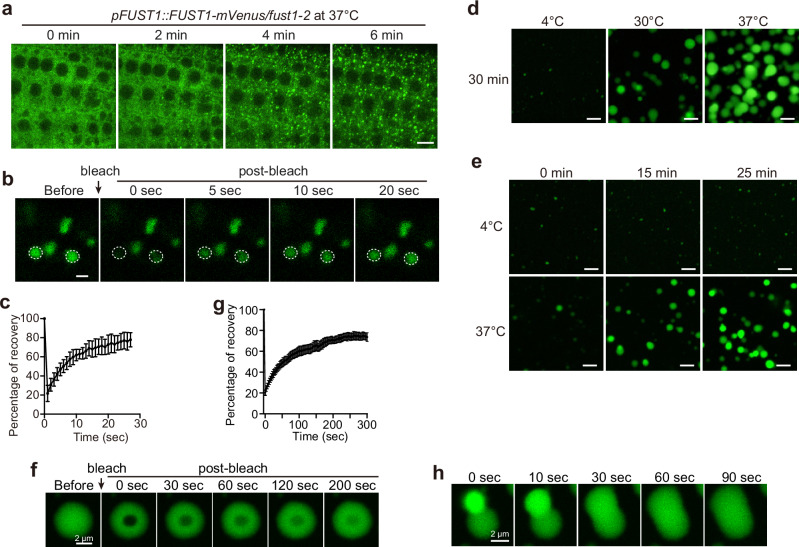
FUST1 undergoes heat-dependent condensation in vivo and in vitro. **a** Time-lapse imaging of FUST1-mVenus condensation in *Arabidopsis* root tip cells. Scale bar, 5 μm. **b** FRAP of FUST1 condensates in *Arabidopsis* root tip cells. Time 0 s indicates the time of the photobleaching pulse. White dashed circles indicate the bleached condensates. Scale bar, 1 μm. **c** Plot showing the fluorescence recovery of FUST1 condensates after photobleaching in *Arabidopsis* root tip cells. Error bars indicate mean ± SD (*n* = 13). **d** In vitro phase separation of 2.5 μM FUST1-GFP at varying temperatures in 40 mM Tris-HCl, pH 7.4 and 100 mM NaCl. Scale bars, 5 μm. **e** Time-lapse imaging of 2.5 μM FUST1-GFP phase separation at 4 °C and 37 °C in 40 mM Tris-HCl, pH 7.4 and 100 mM NaCl in vitro. Scale bars, 5 μm. **f** FRAP of FUST1-GFP droplet. Time 0 s indicates the time of the photobleaching pulse. Scale bar, 2 μm. **g** Plot showing the recovery after photobleaching of FUST1-GFP droplets. Error bars indicate mean ± SD (*n* = 6). **h** Relaxation of two FUST1-GFP droplets into one droplet. Time points are indicated above. Scale bar, 2 μm.

We have previously characterized the potential phase-separation proteins from plants.^20^ These proteins were expressed in tobacco epidermal cells and fission yeast cells and exposed to stress conditions. This allowed us to identify stress-responsive biomolecular condensates in *Arabidopsis*.^21^ AT3G07660 is an uncharacterized protein that we named FUST1, standing for a sacred FuSang Tree in ancient Chinese legend on which ten suns rest. FUST1 was highly enriched as potential phase-separation proteins^20^ and formed cytoplasmic condensates in tobacco epidermal cells in response to heat treatment (Supplementary information, Fig. S1a). FUST1 also formed heat-dependent condensates when heterologously expressed in yeast cells (Supplementary information, Fig. S1b). We obtained two mutant alleles: *fust1-1* harbors a T-DNA insertion within the ninth exon, producing a truncated protein lacking the C-terminal fragment (Supplementary information, Fig. S1c); *fust1-2* is a CRISPR allele that has a deletion of 603-bp fragment harboring promoter, 5’UTR and first exon (Supplementary information, Fig. S1c, e), therefore representing a complete knockout allele (Supplementary information, Fig. S1c‒e). To confirm heat-dependent condensation of FUST1 in vivo, we generated *Arabidopsis* complementation plants expressing the full-length genomic sequence of *FUST1* fused with the coding sequence of a yellow fluorescent protein in *fust1* mutants (Supplementary information, Fig. S1c). The transgenes were expressed at comparable levels to that of endogenous *FUST1* (Supplementary information, Fig. S1d). FUST1-mVenus formed cytoplasmic condensates as quickly as 2 min upon heat stress treatment at 37 °C in the root tip cells (Fig. 1a; Supplementary information, Fig. S1f and Video S1). FUST1 condensates were quickly dissolved by 1,6-hexanediol (Supplementary information, Fig. S1g), dynamically exchanged with the surrounding milieu as probed by fluorescence recovery after photobleaching (FRAP; Fig. 1b, c; Supplementary information, Video S2). FUST1 was ubiquitously expressed in shoot and root tissues as well as in early embryos of imbibed seeds (Supplementary information, Fig. S1h, i). Heat-dependent condensation was also observed in imbibed seeds and leaf epidermal cells (Supplementary information, Fig. S1i, j). We conclude that FUST1 undergoes heat-dependent condensation in vivo.

### FUST1 condensation is intrinsically sensitive to temperature increase

**Fig. 2:**
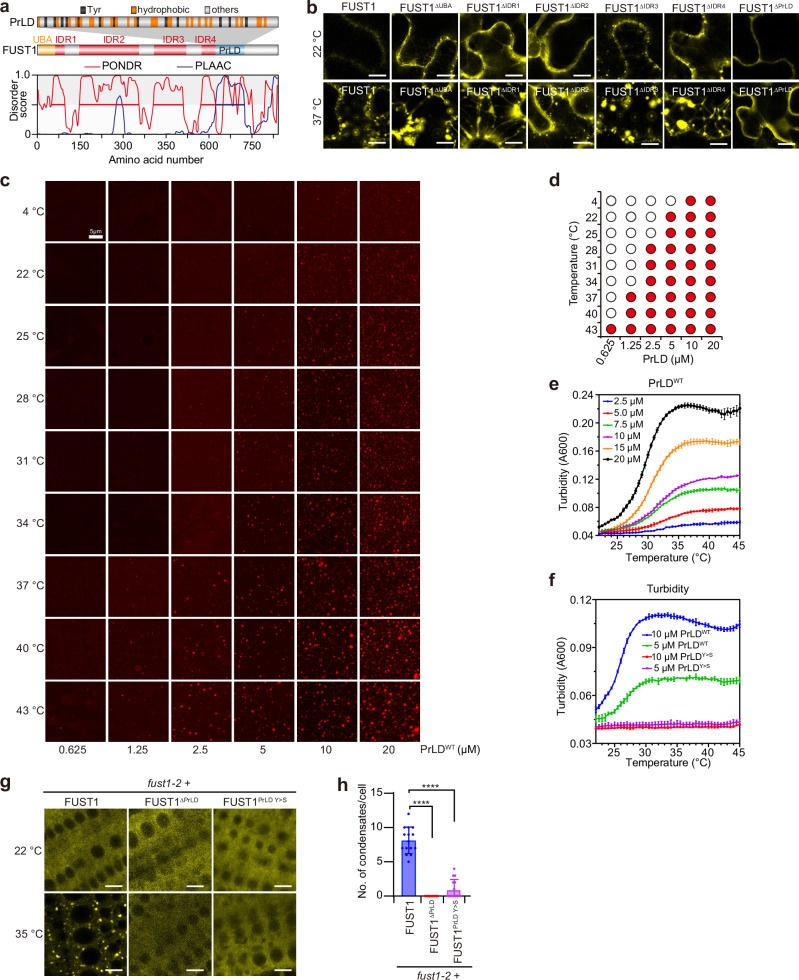
PrLD of FUST1 is the main driver for heat sensing and condensation. **a** Top, illustration of the amino acid composition of PrLD. Middle, protein domain structure of FUST1. Bottom, prediction of the IDRs and prion-like domain by PONDR and PLACC algorithms. **b** Representative confocal microscopic images of tobacco epidermal cells expressing FUST1 and its variants. The cells were treated at 37 °C for 30 min and 22 °C as control. Scale bars, 10 μm. **c** In vitro phase separation assay of Cy5-labeled PrLD^WT^ at different temperatures and concentrations. Scale bar, 5 μm. **d** Phase diagram of PrLD^WT^ as a function of temperature. Red dots, with detectable condensates. Empty circles, without detectable condensates. **e**, **f** Temperature-dependent turbidity of indicated proteins with indicated protein concentrations. In vitro phase separation assay in **c** and turbidity measurements in **e**, **f** were conducted in 40 mM Tris-HCl pH, 7.4 and 500 mM NaCl. Error bars indicate mean ± SD (*n* = 3). **g** Representative confocal microscopic images of *Arabidopsis* root tip cells expressing FUST1, FUST1^∆PrLD^ and FUST1^PrLD Y>S^. The roots were treated at 35 °C for 30 min. Scale bars, 10 μm. **h** Statistical analysis of number of condensates per cell in each genotype in **g** at 35 °C. Error bars indicate mean ± SD (*n* = 15). *P* values were calculated using two-sided Student’s *t*-test. ****P < 0.0001.

FUST1 is largely unfolded (Supplementary information, Fig. S2a) and contains long IDRs (Fig. 2a), promoting us to test whether it undergoes autonomous phase separation. We purified FUST1 that is fused to monomeric GFP from prokaryotic cells (Supplementary information, Fig. S2b) and assessed the phase behavior in vitro. FUST1-GFP formed very few droplets at 4 °C (Fig. 1d, e). Elevating the temperature to 30 °C and 37 °C gradually increased both the size and number of FUST1-GFP droplets (Fig. 1d, e). FUST1-GFP molecules dynamically exchanged between the dense phase and the dilute phase (Fig. 1f, g). Two FUST1-GFP droplets rapidly fused upon contacting each other (Fig. 1h). These features are consistent with the liquid-like property of FUST1 condensates in vivo. Untagged FUST1 also formed more droplets at higher temperature (Supplementary information, Fig. S2c). Temperature-dependent turbidity analysis of FUST1 protein solution confirmed the temperature sensitivity of FUST1 phase separation (Supplementary information, Fig. S2d). To obtain more sensitive measurements, we used dynamic light scattering (DLS) and monitored the apparent hydration radius (Rh), which is a proxy of droplet size, of FUST1 protein solution during a slow temperature ramp. The droplet size of FUST1 grew rapidly between 35 °C and 45 °C (Supplementary information, Fig. S2e), suggesting that FUST1 undergoes a highly cooperative assembly transition when exposed to higher temperatures. To exclude the possibility that FUST1 protein was denatured at elevated temperature, we used far-UV circular dichroism (CD) spectroscopy^22^ to evaluate the secondary structure content. FUST1 protein showed slightly increased CD spectra between 205 nm and 230 nm at higher temperature (Supplementary information, Fig. S2f), which is suggestive of a mild conformational change but not global denaturation. Taken together, these results indicate that FUST1 phase separation is enhanced by heat.

### The heat-dependent condensation of FUST1 is primarily determined by a prion-like domain

FUST1 harbors an N-terminal ubiquitin-associated (UBA) domain, four predicted IDRs and one prion-like domain (PrLD) (Fig. 2a). To define which part(s) of FUST1 is responsible for its condensation, we generated deletion variants and assessed their condensation in tobacco epidermal cells and yeast cells. While variants lacking UBA or IDR still retained condensation to some extent, deletion of PrLD completely abolished FUST1 condensation in both systems (Fig. 2b; Supplementary information, Fig. S3a). Consistently, purified FUST1^∆PrLD^ failed to form condensates in vitro while other variants still did (Supplementary information, Fig. S3b). Furthermore, FUST1^∆PrLD^ lost temperature responsiveness as revealed by the measurement of turbidity and Rh (Supplementary information, Fig. S2d, e).

PrLD contains few positive charges and no negative charge, and is enriched with aromatic and hydrophobic amino acids (Supplementary information, Fig. S3c‒e), consistent with features of lower critical solution temperature (LCST) behavior.^23^ Furthermore, the regularly spaced aromatic tyrosine residues in PrLD may act as attractive stickers.^24^ To test whether PrLD indeed possesses LCST behavior, we purified PrLD without tag fusion to avoid any impact of tagging on its phase behavior (Supplementary information, Fig. S4a) and labeled it with Cy5 for microscopic observation. We found that PrLD formed droplets, which are not destabilized by higher salt concentration (Supplementary information, Fig. S4b). We then characterized the PrLD phase boundary in detail (Fig. 2c, d). At 4 °C, phase separation was observed at protein concentrations above 5.0 μM. As temperature increased, phase separation occurred at lower concentrations, with droplets being observed at concentrations as low as 0.625 μM at 43 °C. These results indicate that the saturation concentration (C_sat_) of PrLD is lowered by increasing temperature, agreeing with an LCST behavior. Consistent with this result, the temperature-dependent turbidity curve of PrLD closely resembled that of a typical LCST protein (Fig. 2e). Conversely, PrLD^Y>S^ lost the temperature-dependent phase separation in vitro (Fig. 2f; Supplementary information, Fig. S4c). In the context of full-length FUST1, neither FUST1^∆PrLD^ nor FUST1^PrLD Y>S^ was able to form condensates, both in vitro and in *Arabidopsis* (Fig. 2g, h; Supplementary information, Fig. S2d). These results indicate that the PrLD of FUST1 is the main driver for heat sensing and condensation.

### PrLD encodes a thermo-switch

**Fig. 3:**
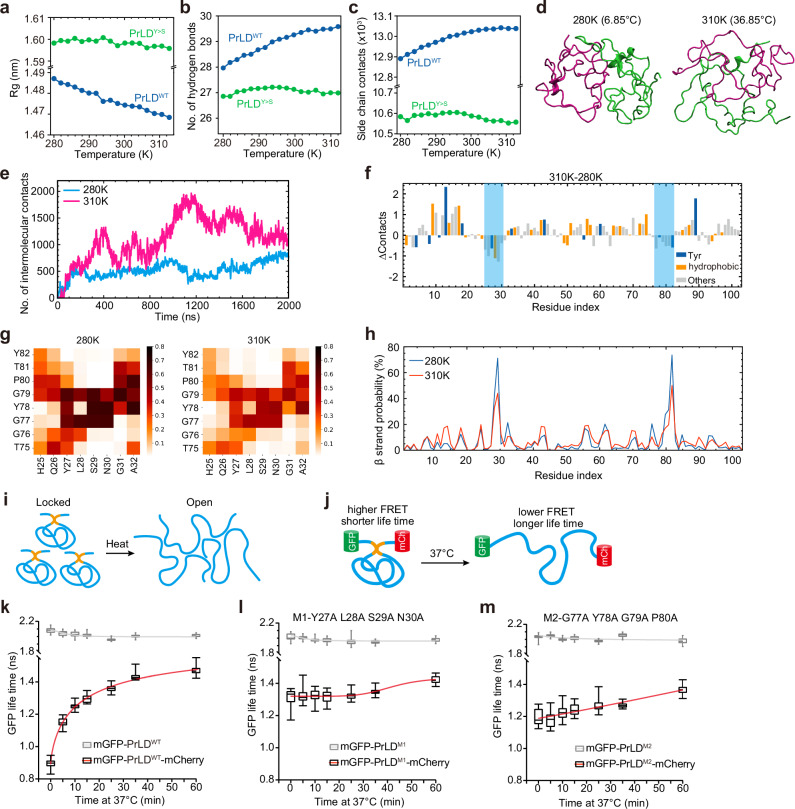
The mechanism of temperature sensing by PrLD. **a**–**c** The gyration of radius (Rg, **a**), the number of intramolecular H-bonds (**b**), and the side chain-side chain contacts between amino acid residues (**c**) of PrLD^WT^ or PrLD^Y>S^ at elevating temperatures as revealed by MD simulation. **d** The conformation of two PrLD molecules at 310 K or 280 K as revealed by MD simulation. **e** The structure prediction shows the intermolecular contacts between two PrLD molecules increase at 310 K or 280 K as revealed by MD simulation. **f** Diagram showing the change of contacts for each amino acid with other residues in PrLD at 310 K compared to 280 K. Positive and negative values indicate gain and loss of contacts, respectively. **g** Heat map showing the strength of contacts between indicated residues at 280 K or 310 K. **h** Diagram showing the β-strand probability across PrLD at 280 K or 310 K. **i** Schematic diagram showing the lock-to-open conformational switch of PrLD at elevating temperatures. The orange region indicates the β-strand lock. **j** Illustration of the FRET assay. **k**–**m** Quantification of mGFP-lifetime in mGFP-PrLD^WT^ or mGFP-PrLD^WT^-mCherry (**k**) mGFP-PrLD^M1^ or mGFP-PrLD^M1^-mCherry (**l**) and mGFP-PrLD^M2^ or mGFP-PrLD^M2^-mCherry (**m**). Error bars indicate mean ± SD (*n* ≥ 20).

To understand the molecular basis underlying how PrLD senses temperature, we carried out MD simulations on PrLD. We used all-atom simulation with explicit water model (Supplementary information, Fig. S5a) to accurately account for the entropy effects of both the solute and the solvent that are important in temperature-responsiveness. The simulation was performed under the CHARMM36m force field, which has improved accuracy in generating conformational ensembles for IDRs.^25^ Further, replica-exchange MD (REMD) was applied with a total simulation time of 62 µs to sample sufficient conformations of the PrLD and its mutant variant (see Methods for details). Our simulations revealed that the radius of gyration (Rg), as a metric of the physical size of the PrLD, decreased as the temperature increased (Fig. 3a), which is a hallmark of LCST behavior.^23^ The same trend was observed for the solvent accessible surface area (SASA) (Supplementary information, Fig. S5b). To investigate the molecular driving force underlying the change of Rg, we analyzed the interactions between amino acids and found that intra-molecular hydrogen bonds (H-bonds, Fig. 3b) and the side chain‒side chain contacts between amino acid residues (Fig. 3c) increased as temperature elevates. It has been reported that the intramolecular contacts at the monomeric level can reflect multivalent intermolecular interactions during phase separation of IDRs.^26,27^ The increase of intramolecular interactions in PrLD therefore suggests enhanced intermolecular affinity. Indeed, we observed more intermolecular contacts (Fig. 3d, e) and intermolecular H-bonds (Supplementary information, Fig. S5c, d) at higher temperature when two PrLD molecules were simulated. As a comparison, the Rg and SASA of PrLD^Y>S^ were much bigger than that of PrLD^WT^ (Fig. 3a; Supplementary information, Fig. S5b) while the number of intramolecular H-bonds and side chain contacts was much less (Fig. 3b, c).

However, when we plotted the difference of contact number between 310 K and 280 K (∆contact) for each residue, we found that while the majority of residues exhibited increased intramolecular contacts, two specific groups of residues (25‒30 and 77‒82, highlighted with blue boxes) exhibited reduced contacts (Fig. 3f). Intriguingly, these residues had strong contacts with each other (Fig. 3g) and together formed high-confident β-strand at 280 K (Fig. 3h; Supplementary information, Fig. S5e). Both the contacts and the β-strand probability reduced at 310 K (Fig. 3g, h). These observations led to the hypothesis that PrLD adopts a locked conformation to prevent intermolecular interactions, which is unlocked by heat, enabling intermolecular interactions thereby phase separation (Fig. 3i).

To test this possibility, we performed Förster resonance energy transfer–fluorescence lifetime imaging (FLIM–FRET) by fusing donor fluorophore mGFP and acceptor fluorophore mCherry to the N and C terminus of PrLD, respectively (Fig. 3j). The lifetime of mGFP can serve as an indicator of lock-to-open conformational switch, as two groups of residues (25‒30 and 77‒82) are positioned near the two ends of the PrLD. The lifetime of mGFP will be short at low temperature and increase upon heating if the proposed conformational switch exists (Fig. 3j). To avoid any impact of protein concentration on FRET efficiency, we quantified donor lifetime only in the dense phases. We found that the lifetime of mGFP in mGFP-PrLD^WT^-mCherry is much lower than the no acceptor control before heating (Fig. 3k), in agreement with a locked conformation. Post treatment at 37 °C, the lifetime of mGFP rapidly increased (Fig. 3k; Supplementary information, Fig. S5f), suggesting open conformations. It should be noted that the reduction of Rg in simulation only indicates the increase of interactions but not the actual conformational change. We then mutated the residues that are involved in the “lock” formation, resulting M1 (Y27A, L28A, S29A, N30A) and M2 (G77A, Y78A, G79A, P80A) variants. FRET analysis showed that both M1 and M2 exhibited higher mGFP lifetime than PrLD^WT^ and showed only minor increase in response to heat (Fig. 3i, m; Supplementary information, Fig. S5f). Consistently, the phase separation of M1 and M2 showed diminished heat responsiveness as observed in both microscopy and turbidity measurements (Supplementary information, Fig. S5g‒i). Taken together, these results indicate that a lock-to-open conformational switch is essential for PrLD of FUST1 to sense temperature.

### FUST1 interacts with and localizes in SGs

**Fig. 4:**
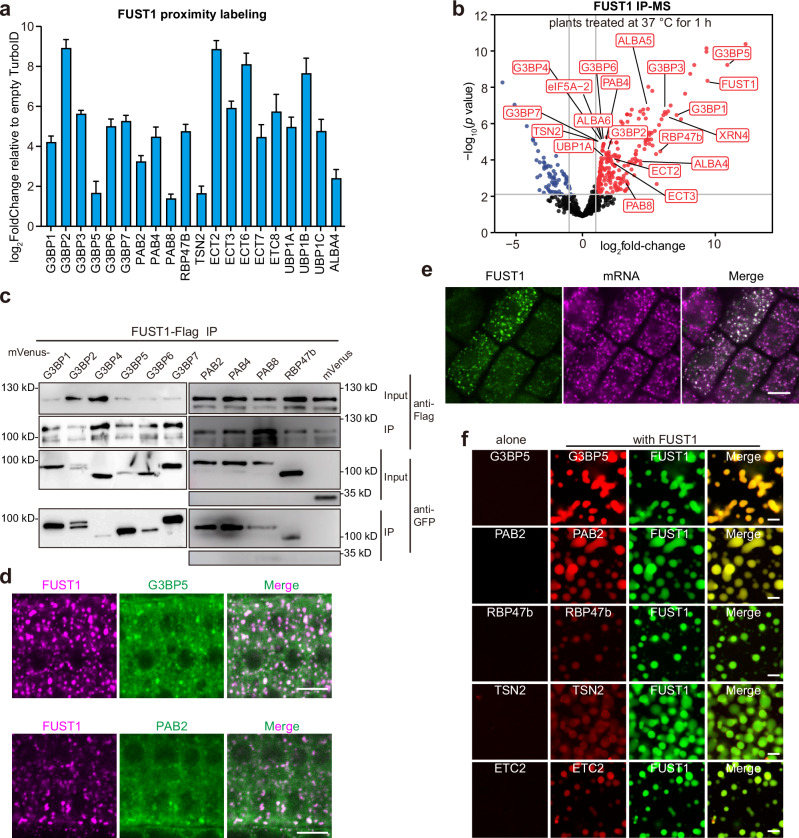
FUST1 interacts with and localizes in the SGs. **a** The integral SG components enriched by FUST1-TurboID proximity labeling. Error bars indicate mean ± SD (*n* = 4). **b** Volcano plot showing the enrichment of proteins by IP-MS of FUST1 in *Arabidopsis*. The values were calculated from 4 biological replicates. **c** Co-immunoprecipitation of FUST1 with indicated proteins expressed in tobacco epidermal cells. Cells were treated at 37 °C for 1 h and crosslinked with UV. Empty vector was included as a negative control. **d** Representative confocal microscopic images of *Arabidopsis* root tip cells co-expressing *FUST1-mScarlet* with *G3BP5-mVenus* or *PAB2-GFP* under their native promoters. Roots were treated at 37 °C for 30 min. Scale bars, 10 μm. **e** FISH with oligodT probes in *pFUST1::FUST1-mVenus/fust1-1* roots treated at 37 °C for 30 min. Scale bar, 10 μm. **f** Partitioning of 2.5 μM indicated proteins by 2.5 μM FUST1 droplets in 40 mM Tris-HCl pH 7.4 and 100 mM NaCl in vitro. Scale bars, 5 μm.

To investigate the function of FUST1 condensates, we sought out to characterize the composition. Given the dynamic property of FUST1 condensates (Fig. 1b), we chose TurboID-based proximity labeling as it allows to capture transient interactions.^28^ *pFUST1::FUST1-TurboID/fust1-1* seedlings were labeled at 37 °C for 1 h (Supplementary information, Fig. S6a). *YFP-TurboID/Col-0* seedlings were treated and labeled in parallel for background control (Supplementary information, Fig. S6a). With four biological replicates, we identified 438 proteins that were enriched at least two-fold by FUST1-TurboID compared to YFP-TurboID (Supplementary information, Fig. S6a and Table S1). Gene Ontology (GO) analysis of these proteins revealed biological functions related to heat stress response and ribonucleoprotein complex biogenesis (Supplementary information, Fig. S6b). Amongst, known integral components of *Arabidopsis* SGs, including G3BPs,^29^ RBP47b,^30^ PABs,^31^ UBP1s,^31^ TSNs,^32^ ECTs,^33^ and ALBA4^34^ were highly enriched (Fig. 4a; Supplementary information, Tables S1, S2). In addition, translation factors, ribosomal proteins, tRNA ligases and chaperons, which were reported to be SG constituents in mammalian cells,^35^ were also highly enriched by FUST1-TurboID (Supplementary information, Tables S1, S2).

To confirm the interactors of FUST1, we performed immunoprecipitation and mass spectrometry (IP-MS) of FUST1 in vivo before and after heat treatment. UV crosslinking was utilized to capture transient protein‒RNA interactions. The results showed that 412 proteins were identified from heat-treated seedlings, including most of the SG components (Fig. 4b; Supplementary information, Table S3). These proteins exhibited similar GO terms to those enriched in TurboID-based proximity labeling assay (Supplementary information, Fig. S6c). The overlapped proteins between FUST1 proximity labeling and IP-MS were mainly involved in stress granule assembly and heat signaling pathway (Supplementary information, Fig. S6e, f). As a comparison of FUST1 IP-MS, fewer SG components were identified in untreated seedlings (Supplementary information, Fig. S6d), suggesting that the interactions with FUST1 are heat-dependent. Some of these interactions were validated by co-immunoprecipitation assay in tobacco epidermal cells (Fig. 4c). Consistent with their interactions, G3BP5 and PAB2 colocalized with FUST1 condensates in stable transgenic *Arabidopsis* (Fig. 4d) and all selected SG markers colocalized with FUST1 when co-expressed in tobacco cells (Supplementary information, Fig. S6g). SGs are cytoplasmic mRNP granules that form from mRNA stalled in translation initiation.^14^ Therefore, polyA mRNA condensates were used as a proxy to represent SGs.^36^ In line with this, fluorescence in situ hybridization (FISH) assay using Cy5-oligo deoxythymidine (dT) probe revealed complete colocalization of FUST1 condensates with mRNAs (Fig. 4e). Additionally, cycloheximide (CHX), which blocks the assembly of SGs,^15^ inhibited FUST1 condensation (Supplementary information, Fig. S6h). Together, these results indicate that FUST1 associates with SGs.

The colocalization of FUST1 condensates with mRNAs promoted us to interrogate the influence of RNA on FUST1 condensation. Addition of low amount of total RNA purified from *Arabidopsis* seedlings strongly promoted the degree of FUST1 condensation in vitro (Supplementary information, Fig. S7a). Both oligodT-enriched mRNA (polyA^+^) and oligodT-depleted RNA (polyA^‒^) enhanced FUST1 condensation as potently as total RNA (Supplementary information, Fig. S7b). Consistent with the in vivo colocalization, FUST1 condensates partitioned RNAs in vitro (Supplementary information, Fig. S7c). We next tested whether FUST1 possesses RNA-binding activity. In the electrophoretic mobility shift assay (EMSA), a 118-nt long RNA of random sequence formed high molecular weight complexes with FUST1 (Supplementary information, Fig. S7e), suggesting non-specificity for FUST1 RNA-binding. A patch towards the N-terminus of FUST1 had a net positive charge density (Supplementary information, Fig. S7d). Indeed, FUST1 with the positive patch deleted (FUST1^∆PP^) failed to form RNA‒protein complexes in the EMSA assay (Supplementary information, Fig. S7e). These results indicate that FUST1 condensates partition mRNAs via electrostatic interactions.

### FUST1 condensation is indispensable for SG assembly

To establish the relationship between FUST1 and SG proteins, we carried out in vitro partitioning assay. We prepared recombinant SG markers including G3BP5, PAB2, RBP47b, TSN2 and ECT2 and found that none of them alone formed condensates in vitro at either low or high temperature (Supplementary information, Fig. S8a). Instead, all of the selected SG proteins were partitioned by FUST1 condensates (Fig. 4f). As negative controls, an RNA-binding protein KH22, which was not enriched by FUST1 proximity labeling, and mCherry were not partitioned by FUST1 condensates (Supplementary information, Fig. S8b, c). Intriguingly, the partitioning of selected SG proteins was dramatically enhanced at 37 °C compared to 4 °C (Supplementary information, Fig. S8b, c). These results suggest that FUST1 could be a driver for SG assembly.

**Fig. 5:**
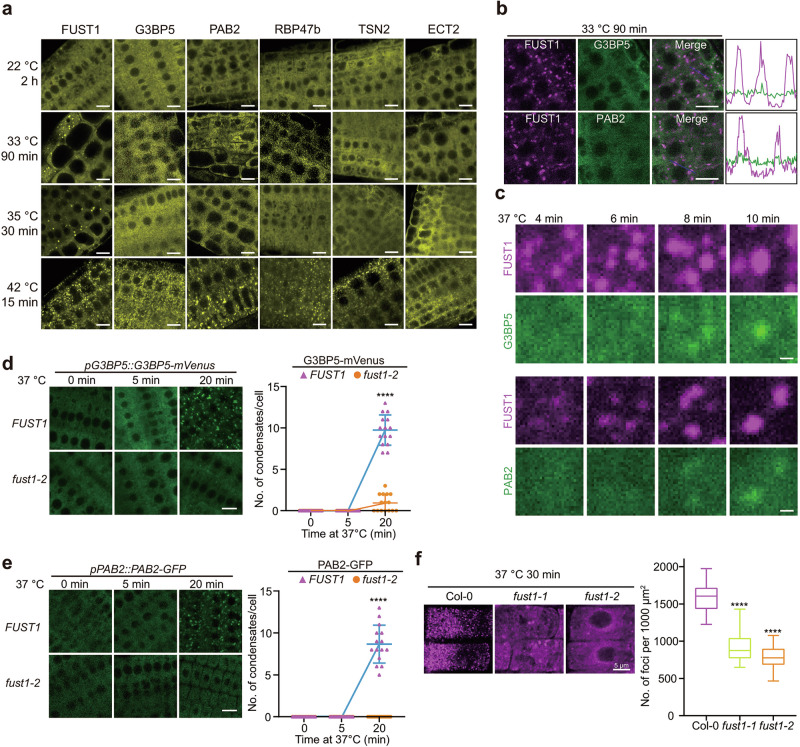
FUST1 condensation is required for SG formation. **a** Confocal microscopy images of *Arabidopsis* root tip cells expressing indicated proteins under the corresponding native promoters. The roots were treated as indicated. Scale bars, 10 μm. **b** Representative confocal microscopic images of *Arabidopsis* root tip cells co-expressing *FUST1-mScarlet* with *G3BP5-mVenus* or *PAB2-GFP* under their native promoters. Roots were treated at 33 °C for 90 min. Scale bars, 10 μm. **c** Time-lapse imaging of *Arabidopsis* root tip cells co-expressing *G3BP5-mVenus* or *PAB2-GFP* with *FUST1-mScarlet* under their corresponding native promoters. The plants were treated at 37 °C for the indicated time. Scale bars, 1 μm. **d**, **e** Left, Confocal microscopic images of *Arabidopsis* root tip cells expressing indicated proteins under corresponding native promoters in FUST1 wild type background or *fust1-2* mutants. The roots were treated at 37 °C for the indicated time. Scale bars, 10 μm. Right, Statistical analysis of the number of condensates per cell in the indicated genotypes. Error bars indicate mean ± SD (*n* = 15). *P* values were calculated using two-sided Student’s *t*-test. ****P < 0.0001. **f** FISH with Cy5-oligodT probes in Col-0 and *fust1* mutants treated at 37 °C for 30 min. Scale bar, 5 μm. *P* values were calculated using two-sided Student’s *t*-test. ****P < 0.0001.

We next sought to determine the hierarchy relationship between FUST1 and SG markers in vivo. In the literature, temperatures at or above 37 °C and duration of treatment longer than 10 min were used to induce G3BP5-,^37^ PAB2-,^30^ RPB47b-,^38^ TSN2-,^32^ and ECT2-labeled^33^ SGs. We tested this in stable transgenic plants expressing each of the SG markers under the corresponding native promoter. We confirmed that condensation of SG markers required temperatures above 35 °C and at least 10 min (Fig. 5a; Supplementary information, Fig. S9). In contrast, FUST1 condensation occurred below 35 °C and as quickly as 2 min at 37 °C (Fig. 5a; Supplementary information, Fig. S9), suggesting that condensation of FUST1 and SGs is a sequential event with FUST1 being earlier than SG formation.

To more accurately compare the dynamics of FUST1 with SG components during condensation, we co-expressed *FUST1-mScarlet* transgene with *G3BP5-mVenus* or *PAB2-GFP* transgene and performed double-colored time-lapse live imaging in the same root cells. The results revealed that below SG threshold temperature, FUST1 condensation occurred autonomously without G3BP5 or PAB2 (Fig. 5b), and above SG threshold temperature, FUST1 condensates formed first and G3BP5 or PAB2 was subsequently condensed into FUST1 condensates (Fig. 5c). These observations prompted us to test whether FUST1 is required for heat-induced SG assembly. Herein, we crossed *fust1-2* mutant into the SG marker lines and found that the formation of PAB2, and G3BP5-labeled SGs was drastically delayed in *fust1-2* background (Fig. 5d, e). In line with this, FISH assay revealed that polyA RNA condensates were drastically reduced in *fust1* mutants (Fig. 5f). Together, these data demonstrate that FUST1 condensation precedes condensation of several known SG markers and is indispensable for SG assembly.

### FUST1 confers both basal and acquired heat tolerance

**Fig. 6:**
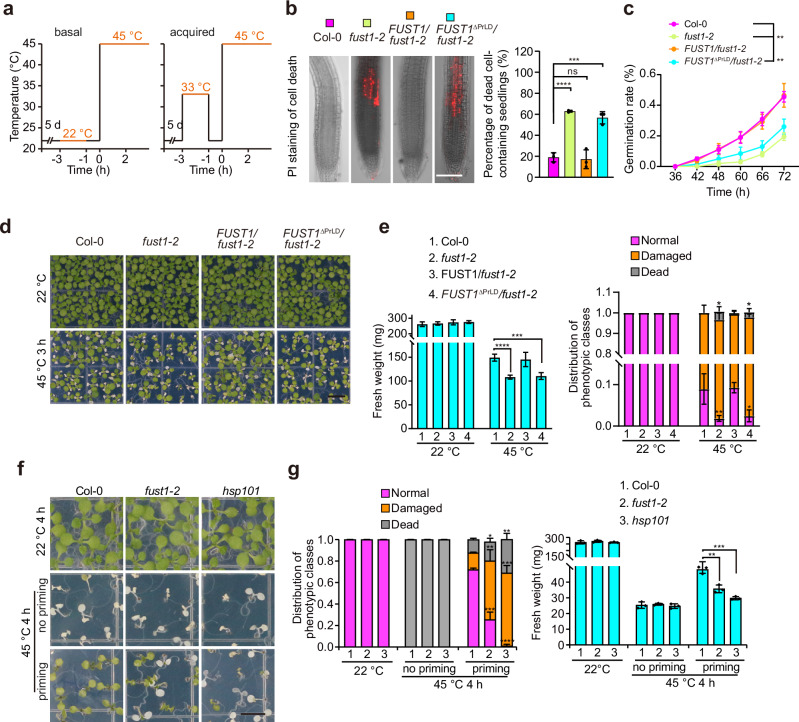
FUST1 is required for thermotolerance. **a** Scheme of heat exposures in basal and acquired tolerance assays. **b** Left, cell death staining of *Arabidopsis* roots upon heat stress treatment at 42 °C for 2 h. Scale bars, 100 μm. Right, the percentage of seedlings containing cell death. Error bars indicate mean ± SD (*n* = 3). Twenty seedlings were assayed in each replicate. *P* values were calculated using two-sided Student’s *t*-test. ***P < 0.001, ****P < 0.0001. ns no significance. **c** Germination rate of *Arabidopsis* seeds after heat treatment at 50 °C for 60 min. Error bars indicate mean ± SD (*n* = 4). Each replicate contains 49 seeds. *P* values were calculated using two-sided Student’s *t*-test. **P < 0.01. **d**, **f** Phenotypes of indicated seedlings assayed for basal (**d**) or acquired (**f**) heat tolerance. Pictures were taken at seven days (**d**) or four days (**f**) of recovery after treatment as indicated. Scale bars, 0.5 cm. **e**, **g** Quantification of the damage rate and fresh weight of seedlings shown in **d**, **f**. Error bars indicate mean ± SD (*n* = 3). At least 50 seedlings were assayed for each replicate. *P* values were calculated using two-sided Student’s *t*-test. *P < 0.05, **P < 0.01, ***P < 0.001, ****P < 0.0001.

To assess the functional significance of FUST1 condensation, we first examined the basal thermotolerance (Fig. 6a). Five-day-old seedlings were subjected to heat shock at 45 °C for 2 h and stained with propidium iodide (PI) to assess cell viability. While the majority of Col-0 plants showed no PI signal, *fust1* mutants had a much higher frequency of PI staining and this defect was fully recovered by FUST1 re-expression (Fig. 6b; Supplementary information, Fig. S10a), suggesting that FUST1 is necessary for early heat signaling events that confer cell survivability. And we observed the germination ability of *fust1* mutant seeds. Seeds of *fust1-2* showed decreased germination rate upon heat treatment, whereas we rescued this phenotype by expressing FUST1, while the germination rate was similar to Col-0 under normal condition (Fig. 6c; Supplementary information, Fig. S10b). To assess the phenotypes at tissue level, seedlings were recovered at normal growth temperature for 7 days after heat treatment to allow cell division and elongation. Compared to Col-0, *fust1* mutants had significantly higher fraction of pale or white sectors on leaves that are indicative of cell death and exhibited retarded growth and less fresh weight (Fig. 6d, e; Supplementary information, Fig. S10c). We next performed prolonged heat stress by placing five-day-old seedlings under 37 °C for 2 days. Post recovery for 5 days, we observed that root growth of *fust1-1* seedlings was much slower than Col-0 (Supplementary information, Fig. S10d). The defect of *fust1* mutant in heat tolerance can be fully rescued by expressing full-length FUST1 but not by the variant lacking PrLD (Fig. 6b‒e; Supplementary information, Fig. S10d), further supporting the importance of PrLD for FUST1 function. These data indicate that FUST1 is required for basal thermotolerance.

Plants acquire enhanced tolerance to stress through prior exposure to a milder challenge, a process known as defense priming.^39^ Given that FUST1 formed condensates in response to mild temperatures, we tested whether FUST1 is necessary for acquired heat tolerance. Seedlings were exposed to a priming period at 33 °C for 2 h followed by a recovery phase for 1 h (Fig. 6a) and subsequent heat shock at 45 °C for 4 h. While almost all seedlings of both wild type and mutant died without priming, over 90% of wild-type Col-0 seedlings survived after priming (Fig. 6f, g), indicating that wild-type seedlings acquired increased thermotolerance upon priming at 33 °C. In contrast, more than 70% of the *fust1-2* seedlings either developed severely damaged leaves or completely died even after priming (Fig. 6f, g), indicating that the thermotolerance acquired by pre-treatment at 33 °C is FUST1-dependent. Consistent with the essential role of molecular chaperone HSP101 in acquired thermotolerance,^40^ the majority of *hsp101* mutant plants died after priming (Fig. 6f, g). Measurement of the fresh weight of those seedlings confirmed this trend (Fig. 6g). These results demonstrate that FUST1 is indispensable for acquired thermotolerance.

### Temperature-dependent condensation of FUST1 is conserved in plants

To assess whether the condensation of FUST1 is conserved in plants, we explored the diversity of this protein in other plant species. FUST1 homologs were found in all land plants but not in green algae (Supplementary information, Fig. S11a). Multiple sequence alignment of FUST1 homologs from angiosperms revealed that conservation score of the PrLD was much higher than other IDRs (Supplementary information, Fig. S11b). In particular, the tyrosine residues, which are key for heat-dependent phase separation, were highly conserved (Supplementary information, Fig. S11c). The sequence comprising the β-strand lock is also conserved (Supplementary information, Fig. S11b, c), suggesting that “lock-to-open” mechanism for heat sensing in PrLD is preserved during evolution. We expressed FUST1 of maize, soybean and Chinese cabbage in tobacco epidermal cells and found that they all exhibited heat-dependent condensation (Supplementary information, Fig. S11d). Like *Arabidopsis* FUST1, in vitro phase separation of all homologs was heat-dependent as revealed by DLS assay (Supplementary information, Fig. S11e). These results suggest that FUST1 is functionally conserved in plants.

## Discussion

**Fig. 7:**
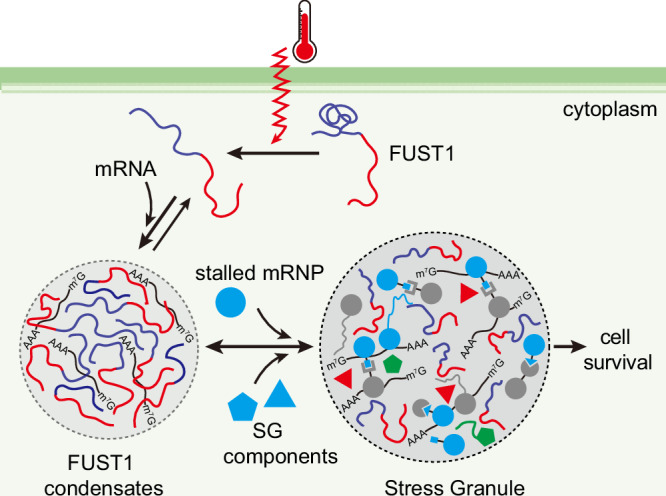
A proposed model for FUST1 in heat sensing and response. In the cytoplasm, FUST1 is dispersed. When the temperature increases, FUST1 senses heat via conformational switch and condenses with RNA. FUST1 condensates provide primers to recruit stress granule components and drive stress granule assembly, which is required for cell survival.

In summary, our findings demonstrate that an uncharacterized protein, FUST1, directly senses elevated temperature, giving rise to heat-dependent condensation in *Arabidopsis*. Notably, FUST1 condensation precedes condensation of several known SG markers, priming the assembly of SGs thereby supporting cell survival under heat stress (Fig. 7). The combination of all-atom MD simulations and biophysical analyses allow us to uncover the mechanism of thermosensing by FUST1. The PrLD has a strong ability to drive phase separation which is autoinhibited via a β-strand lock under low temperature. Increasing temperature tends to break the β-strand, allowing intermolecular interactions to occur and releasing phase separation. Our data also indicate that the sticker tyrosine residues are crucial for both phase separation and heat-dependent conformational rearrangements, but the spacer residues, in particular the hydrophobic ones, also make key contributions. Future work should pinpoint the sequence grammar for thermosensing. Heat-induced conformational changes were reported recently for a transcriptional co-regulator TWA1 in *Arabidopsis*^10^ and translation initiation factor eIF4G in yeast,^11^ resulting in changes of interaction partners and condensate formation. *Arabidopsis* EARLY FLOWERING 3 (ELF3), a component of the evening complex, senses heat via its PrLD^8^ with the mechanism being obscure. The atomic simulation and biophysical approaches established in this study will shed light on understanding the molecular basis of heat sensing by both globular and disordered proteins.

Further, autoinhibition mechanism was also described for G3BP phase separation during mammalian SG formation. In this scenario, two IDRs within G3BP interact, giving rise to a more closed conformation that reduces its valency. When RNA reaches a certain threshold, it competes with IDR for interactions, disrupting intramolecular interactions and allowing the protein to undergo RNA-dependent phase separation.^16,18^ We envisage that autoinhibition could be a general strategy in the regulation of phase separation. While extensive studies have focused on the electrostatics and multivalent weak interactions at the amino-acidic level, our work suggests that dynamic secondary structure, such as β-strand, could be an overlooked yet crucial feature for specific IDR sequences in driving and modulating phase separation.

According to the studies in mammalian cells, SGs are further separated into internal structures including cores and shells.^35,41^ The cores are more dense and solid while the shell is less concentrated and more dynamic. It has been under debate how the substructures in SGs are assembled. Two models, “core first” model and LLPS first model, were postulated.^14^ We propose that FUST1 forms the shell network that allows the “cores” to assemble. The interactions between FUST1 and the “cores” are weak and transient, making the SG dynamic. Indeed, biochemical purification of the solid-like “cores” of SG from *Arabidopsis* identified proteins including SG markers we used in this study but not FUST1.^38^ Future investigation of FUST1 should help us to understand the exact mechanism of SG assembly.

*Arabidopsis* acquires thermotolerance mostly by priming at 37 °C or higher,^10,42^ which induces the production of signals including but not limited to heat shock proteins,^39^ epigenetic factors,^43^ metabolites^44^ that can last for short period of time. We show that a milder temperature at 33 °C also acquires increased heat stress tolerance in a FUST1-dependent manner, implying that biomolecular condensates could be a novel mechanism for short-term memory that enables plant acclimation to stress. Due to its dynamicity and reversibility, we propose that FUST1 condensates act as an alarm system that warns the plants of upcoming severe heat stress.

## Materials and methods

### Plant materials and growth conditions

The T-DNA insertion mutants *fust1-1* (SALK_116148C) and *hsp101* (SALK_099583C) were obtained from the AraShare. The *pPAB2::PAB2-GFP/pab2/8* transgenic line was described previously.^45^ Seeds were sterilized and sown on half-strength Murashige and Skoog (MS, PhytoTech, M519) medium with 1.0% sucrose, 0.4% phytagel (Sigma, P8169) at pH 5.8. Plate media were stratified at 4 °C for 2 d and transferred to a growth chamber under long-day condition (16-h light at 22 °C and 8-h darkness at 18 °C).

### DNA constructs

To generate pFUST1::FUST1-mVenus, pRBP47B::RBP47B-mVenus, pG3BP5::G3BP5-mVenus, pECT2::ECT2-mVenus and pTSN2::TSN2-mVenus constructs, genomic DNA fragments including the promoter regions of approximately 2 kb were amplified from wild-type Col-0 genomic DNA and cloned into the pCAMBIA1300-N1-mVenus vector (digested with *Hind*III). To generate pFUST1::FUST1^∆PrLD^-mVenus constructs, *FUST1* genomic sequence lacking PrLD-coding region was generated and inserted into pCAMBIA1300-N1-mVenus vector (digested with *Hind*III). All the constructs were introduced into *Agrobacterium tumefaciens* strain GV3101 and transformed into *fust1* mutants or wild-type Col-0 plants via the standard floral dipping method. Positive transformants were selected on half-strength MS plates containing 30 mg/L hygromycin (AMRESCO, K547). Homozygous transgenic lines were used for experiments.

The construct for generating *fust1-2* CRISPR mutant was based on CRISPR-Cas9 system reported previously.^46^ Two sgRNAs were designed for *FUST1*, and inserted into the *Bbs*I sites of the pAtU6-26-M vector. The primers used for annealing are listed (Supplementary information, Table S4). The Cas9 cassettes were subcloned into *Kpn*I and *EcoR*I sites of pCambia1300-UBQ: Cas9-P2A-GFP-rbcS-E9t.^46^

The constructs for expression in tobacco epidermal leaf cells were cloned by inserting the coding sequences into the pCAMBIA1300-35S-N1-mVenus, pCAMBIA1300-35S-N1-Flag or pCAMBIA1300-35S-N1-mScarlet vector (digested with *Kpn*I).

To generate the constructs used for heterologous expression in yeast cells, the coding sequences of *FUST1* and its variants were amplified and inserted into the pDUAL-Pnmt1-yeGFP vector (digested with *Nhe*I and *Bam*HI).

To generate the constructs used for in vitro protein expression, coding sequences were amplified and inserted into the pET11-6× His-GFP, pET11-6× His-mCherry (digested with *Nhe*I and *Bam*HI) or pET11-6× His expression vector (digested with *Nhe*I and *Xho*I). Where necessary, a maltose-binding protein (MBP) solubility tag was placed at the N-terminus of the construct, followed by a tobacco etch virus (TEV) protease cleavage site.

All cloning was performed using the ClonExpress II One Step Cloning kit (Vazyme, C112). Primer sequences are provided (Supplementary information, Table S4).

### Quantitative RT-PCR analysis

Total RNA was extracted from *Arabidopsis* seedlings using TRIzol reagent (Invitrogen, 15596018) according to the manufacturer’s protocol. Contaminating DNA was removed using DNase I (Promega, M6101). Reverse transcription was performed by M-MLV reverse transcriptase (Invitrogen, 28025013) and by oligo(dT) primer. Quantitative PCR reactions were performed using the Applied Biosystems^®^ 7500 fast with 2× M5 HiPer SYBR Premix EsTaq (Mei5 Biotechnology, MF787-T) in a final volume of 20 μL. *Tubulin* was used as an internal control. The primers used for PCR are listed (Supplementary information, Table S4).

### GUS staining

Ten-day-old *pFUST1::GUS*/Col-0 seedlings were immersed in the GUS staining solution (0.5 mg/mL X-glucuronide in 100 mM sodium phosphate pH 7.0, 0.5 mM ferricyanide, 0.05 mM ferrocyanide, 1 mM EDTA, and 0.1% Triton X-100) for 12 h at 37 °C. After staining, the seedlings were washed several times in 75% (v/v) ethanol to clear chlorophyll.

### Yeast transformation for heterologous expression

The plasmids were linearized with *Not*I and the fragments were gel-purified and transformed into the fission yeast strain LD328 (genotype *his3-D1 leu1-32*) as described previously.^47^ Briefly, yeast cells were cultured until the OD_600_ reached 0.4‒0.8. For each reaction, 500 µL cultured cells were collected, washed three times with sterilized water and resuspended in buffer I (240 µL of 50% PEG3350, 36 µL of 1.0 M LiAc and 50 µL of 2.0 mg/mL carrier DNA). The linearized DNA (34 µL, up to 1 µg) was added to the resuspended cells, mixed vigorously and incubated at 42 °C for 40 min. The cells were collected and resuspended in 100 µL water and plated on EMM + HT (EMM medium supplemented with 45 mg/L histidine and 15 µM thiamine) plates. After incubation at 30 °C for 2‒3 days, individual colonies were selected on EMM + H (EMM medium supplemented with 45 mg/L histidine) plates. The cells were used for subsequent imaging analyses.

### Fluorescent imaging of cells and tissues

Vertically grown *Arabidopsis* seedlings or a tobacco leaf were soaked in liquid half-strength MS medium and treated at the indicated temperatures for the indicated time. The root tip or a small leaf disc was mounted on a slide, covered with a coverslip and immediately imaged under a Zeiss LSM880 confocal microscope. GFP was excited at 488 nm and detected at 491‒535 nm. mVenus was excited at 514 nm and detected at 529‒570 nm, mScarlet or mCherry was excited at 561 nm and detected at 575‒625 nm. The signal of mVenus and mScarlet was acquired sequentially to avoid cross contamination.

For time-lapse imaging of FUST1 condensation, four-day-old vertically grown *Arabidopsis* seedlings were embedded with solid half-strength MS medium in 15 mm Glass Bottom Cell Culture Dish (NEST, 801002). The dish was imaged at 37 °C on Nikon AX R confocal microscope equipped with Okolab microscope incubator using 40× 0.95-NA objective. Excitation and emission of mVenus and mScarlet were as mentioned above. The interval of image acquisition is 1 min.

For imaging of yeast cells, a colony grown on a medium plate was inoculated into liquid medium and cultured overnight (OD_600_ < 1.5). Ten microliters of cells were collected by centrifugation and resuspended in liquid medium with or without heat treatment. The cells were sprayed onto a slide and covered with a coverslip. Imaging was performed on a Zeiss LSM880 with Airyscan (AxioObserver 7) mode confocal laser microscope. GFP was excited as mentioned above.

### Protein expression and purification

All proteins were expressed in the *Escherichia coli* Rosetta strain. Cells were cultured at 37 °C until the OD_600_ reached 0.4 and induced with 0.5 mM isopropyl-β-d-thiogalactopyranoside (IPTG) at 16 °C for 16‒20 h. Cells were collected, resuspended in lysis buffer (40 mM Tris-HCl pH 7.4, 500 mM NaCl, 10% glycerol) and sonicated for lysis. The lysates were centrifuged at 15,000 rpm for 60 min at 4 °C and the supernatants were passed through Ni-NTA column (Smart-Lifescience, SA004100). The bound proteins were washed with wash buffer (40 mM Tris-HCl pH 7.4, 500 mM NaCl, 20 mM imidazole), eluted with elution buffer (40 mM Tris-HCl pH 7.4, 500 mM NaCl, 500 mM imidazole) and further purified on a Superdex 200 Increase 10/300 column (GE Healthcare, 28990944). Proteins were stored in storage buffer (40 mM Tris-HCl pH 7.4, 500 mM NaCl, 1 mM DTT). The quality of purified proteins was determined by SDS-PAGE and Coomassie blue staining.

### Fluorophore labeling of proteins

Fluorescent labeling dye Cy5 NHS esters (AAT Bioquest) were dissolved in DMSO as a stock at the concentration of 10 mg/mL. The dye was added to the purified protein in 20 mM HEPES, pH 7.5, 500 mM NaCl, 1 mM DTT to the final concentration of 10 µg/mL. The labeling was carried out with gentle rotation at 4 °C overnight in the dark, and then quenched by adding 200 mM Tris-HCl. The unbound dye was removed using three concatenated 5 mL HiTrap desalting columns (GE Healthcare, GE17-1408-01).

### In vitro phase separation assay

For in vitro phase separation, the solubility tag MBP was removed by TEV protease. TEV cleavage efficiency was confirmed by SDS-PAGE analysis. The proteins were diluted to desired concentrations with indicated ionic strengths. The protein samples were incubated at indicated temperatures for indicated time before imaging. For the in vitro phase separation assay of Cy5-labeled PrLD^WT^ at different temperatures (Fig. 2c), proteins were diluted to desired concentrations in 40 mM Tris-HCl pH 7.4 and 500 mM NaCl. Each image represented an independent experimental result, and the protein was incubated at the indicated temperature for 15 min before imaging. To analyze the effect of RNA on FUST1 phase separation, various concentrations of *Arabidopsis* total RNA, polyA^+^ RNA or polyA^‒^ RNA were added to the protein samples in 40 mM Tris-HCl pH 7.4, 100 mM NaCl. RNAs were visualized by GelRed (Mei5 Biotechnology, MF079-plus-01). Droplets were imaged in a 384-well, low-binding multiwell 0.17-mm microscopy plate (Greiner bio-one, 781090) using a Zeiss LSM880 confocal microscope equipped with a 63× 1.40-NA oil objective. The quantification of mean intensity was performed in ImageJ.

### FRAP assay

FRAP of FUST1 condensates in *Arabidopsis* root cells was performed on a Zeiss LSM880 confocal laser microscope. After 5 acquisitions, ROI(s) corresponding to FUST1 condensate(s) was bleached with 5 iterations using a laser intensity of 100% at 488 nm. mVenus fluorescence was detected using a 63× 1.40-NA oil objective. Recovery was recorded for every second for a total of 30 s after bleaching.

In vitro FRAP of FUST1 droplet was carried out with protein samples in 384-well microscopy plates using a Zeiss LSM880 confocal laser microscope equipped with 100× oil immersion objective. A small area of the droplet was bleached with 5 iterations of 100% laser intensity at 488 nm. After bleaching, recovery was recorded for every second for a total of 150 seconds.

### Turbidity measurements

The proteins were diluted to desired concentrations in 40 mM Tris-HCl pH 7.4, 100 mM NaCl or 500 mM NaCl. The protein samples of 100 µL were transferred into flat bottom 96-well plates (Corning, 3364) on ice. Temperature-dependent turbidity measurements were performed on VARIOSKAN FLASH (Thermo) with temperature increasing from 22 °C to 45 °C at a rate of 0.5 °C/min. The absorbance at 600 nm or 350 nm was recorded at each temperature point.

### DLS measurements

The proteins were diluted to desired concentrations in 40 mM Tris-HCl pH 7.4, 100 mM NaCl. The protein samples of 200 µL were transferred into quartz cuvette (WYATT, JC-0247). DLS measurements were performed using DynaPro NanoStar (Wyatt). For data collection, each time point was the average of three 0.25 s acquisitions filtering out samples with a baseline higher than 1.003. The temperature was set to increase at the speed of 1 °C/min. Measurements were recorded every minute. Data were analyzed in the DYNAMICS software with a cumulant fit to the autocorrelation function. Protein concentrations are specified in the Figure legend.

### CD spectroscopy

CD measurements were performed on a Chirascan plus (Aimil) using 1 mm path length cuvette. The FUST1 protein was diluted to a final concentration of 0.1 mg/mL (1.07 µM) in 40 mM Tris-HCl pH 7.4, 50 mM NaCl. CD spectra were recorded from 260 nm to 200 nm with a bandwidth of 1 nm, a 0.5 s integration time with data collected every 1 nm. Each data point was the average of 3 scans. Spectra were baseline corrected using buffer.

### FLIM-FRET

Protein samples were incubated with 6% PEG6000 in 40 mM Tris-HCl, pH 7.4 and 100 mM NaCl to facilitate droplet formation in 384-well microscopy plates. FLIM-FRET was performed using a Leica TCS SP8 laser-scanning confocal microscope equipped with a 100×/1.40-NA oil immersion objective. Samples were scanned with a slow speed of 100 Hz with a repetition rate of 80 MHz. mGFP fluorescence was excited at 488 nm and detected at 500‒540 nm using the in-built hybrid detector. A time-correlated single photon counting (TCSPC) system was used for recording photon events.

All FLIM data analysis was performed using Leica LAS X FLIM FCS software. The minimum threshold count of recorded photons for modeling was 100. The recorded TCSPC photon arrival time histogram of mGFP-PrLD^WT^-mCherry or mGFP-PrLD^M1/2^-mCherry showed multi-exponential decay. Therefore, the photon arrival times were fitted to a double-exponential reconvolution function, allowing the calculation of mean lifetime by intensity weight. A minimum of 20 ROIs were selected for analysis.

### All-atom MD simulation

The initial structures of the wild-type and the mutant (Y > S) PrLD were predicted by the AlphaFold2 program^48^ based on their amino acid sequence. The protein was put into a simulation box with the dimensions of 7 × 7 × 7 nm solvated with explicit water molecules. Chloride ions were added to make the simulation system electrostatically neutralized. The final simulation system contains approximately 34,000 atoms. The protein was described by the CHARMM36m force field. The TIP3P model^49^ was used to represent water molecules. After energy minimization with the steepest descent method, a 200 ns MD simulation at a pressure of 1 bar and temperature of 323 K was performed. From the last 100 ns trajectory, 62 simulation systems with different protein conformations were randomly extracted for the REMD simulations^50^ with the temperature ranging from 280 K to 316 K. 500 ns MD sampling simulations under NPT ensemble were subsequently performed for each replica. Exchanges between two replicas were attempted every 2 ps, resulting in a mean acceptance ratio of 20%. In addition, 2 µs conventional MD simulation was carried out to investigate the intermolecular interactions between two PrLD at 280 K and 310 K, respectively.

All simulations were performed using the Gromacs 2019 package.^51^ The pressure was maintained at 1 bar using the Parrinello-Raham barostat^52^ with a relaxation time of 2.0 ps, the temperatures were kept at the chosen values using the velocity rescaling method^53^ with a coupling constant of 0.1 ps. Bonds containing hydrogen were constrained by the LINCS algorithm.^54^ The non-bonded interactions were calculated at a cutoff distance of 1.0 nm, the particle mesh Ewald (PME) method^55^ with a cutoff radius of 1.0 nm was employed to treat the long-range electrostatic interactions. The equations of motion were integrated using a leap-frog algorithm with a time step of 2 fs. 3D periodic boundary conditions were applied to all the simulations.

The protein structures sampled by REMD simulations were characterized by Rg, intramolecular H-bond, side chain-side chain contacts, solvent accessible surface areas and beta-strand probability. The data analysis was based on simulations from 100 to 500 ns, with the first 100 ns of REMD trajectories discarded to avoid bias from the starting conformation. The formation of side chain‒side chain contact was defined if the minimum distance between two residue side chains is less than 0.6 nm. Intramolecular contacts were ignored between three adjacent residues. The interactions between two PrLD molecules were characterized by intermolecular H-bonds and contacts, and the last 1 µs simulation data were used as statistical averages. H-bonds were defined based on distance and angle with the acceptor-donor distance being within 0.35 nm and the acceptor-donor-hydrogen angle being less than 30°. The beta-strand potential was calculated with the DSSP method.^56^ All trajectory analyses were accomplished using our in house developed scripts and tools implemented in the Gromacs and MDTraj packages.^57^ For structural visualization and animation production, the Visual Molecular Dynamics (VMD) program was used.^58^

### Assays for heat stress tolerance

For basal tolerance analysis, five-day-old *Arabidopsis* seedlings grown horizontally on half-strength MS plates were treated at 45 °C for 3 h and recovered at 22 °C for 7 days in the growth chamber. Photographs were taken and the root lengths were measured.

For the acquired heat tolerance response, five-day-old *Arabidopsis* seedlings grown either horizontally or vertically on half-strength MS plates were treated at 33 °C for 2 h, recovered at 22 °C for 1 h and then treated at 45 °C for 4 h in the growth chamber. As no priming control, seedlings from the same batch were treated in parallel except for 33 °C treatment. The seedlings were then assayed for survival rate, fresh weight or root length. Photographs were taken and the damage rates were analyzed after recovery at 22 °C for 4 days.

### Seed germination assays

For germination analysis, about 50 sterilized seeds were treated at 50 °C or 22 °C for 1 h in distilled water, placed onto half-strength MS medium plates and incubated in a growth chamber under long-day condition (16-h light at 22 °C and 8-h darkness at 18 °C). Testa and endosperm rupture were photographed over time through a Stereo Microscope (Nikon, SMZ18).

### PI staining

To detect cell death of *Arabidopsis* seedlings after heat treatment, PI staining was performed. Five-day-old seedlings were treated at 42 °C for 2 h in ½ MS liquid medium. Roots were subsequently stained with 10 μg/mL PI (MedChemExpress, HY-D0815) for 5 min at room temperature, washed once in distilled water and imaged under Zeiss LSM880 confocal microscope. The excitation and emission wavelengths are 561 nm and 591‒635 nm, respectively. The ratio of seedlings with PI staining was calculated.

### Immunoprecipitation with UV crosslinking

Seven-day-old *pFUST::FUST1-mVenus/fust1* and Col-0 seedlings grown on half-strength MS plates were treated with or without heat stress at 37 °C for 1 h. Seedlings were immediately crosslinked by 600 mJ/cm^2^ UV in cold phosphate buffer saline (PBS) buffer (pH 7.4). After crosslinking, 2 g seedlings were fast-frozen and ground in liquid nitrogen. The resulting fine powder was resuspended in 2 mL of lysis buffer (20 mM Tris-HCl, pH 7.4, 150 mM NaCl, 4 mM MgCl_2_, 0.5% NP-40, 5 mM DTT, 1× Protease Inhibitor Cocktail) and incubated at 4 °C for 30 min with rotation. After filtering by Miracloth (Millipore, 475855-1RCN), the supernatant was incubated with 10 μL of GFP-Nanoab-Magnetic Beads (LABLEAD, GNM-25-1000) for 30 min at 4 °C. The beads were washed four times with lysis buffer and then boiled in SDS loading buffer. Four biological replicates were performed for this experiment.

### Proximity labeling with Turbo-ID

Ten-day-old seedlings of *35S::YFP-TurboID/*Col-0 and *pFUST1::FUST1-TurboID/fust1-1* were treated at 37 °C for 1 h in half-strength liquid MS medium containing the final concentration of 50 μM biotin (Invitrogen, B1595). The seedlings were washed three times with distilled water and flash-frozen with liquid nitrogen. The tissues were ground to fine powder and resuspended with 2 mL ice-cold extraction buffer (50 mM Tris-HCl, pH 7.5, 150 mM NaCl, 0.1% SDS, 1% Triton X-100, 0.5% sodium deoxycholate, 1 mM EDTA, 1 mM DTT, 1 mM PMSF, 1× Protease Inhibitor Cocktail) per gram powder. Lysates were centrifuged twice at 4 °C for 10 min and the supernatant was passed through 0.22 μm filter (Millipore, SLGVR33RB).

To remove free biotin, extract was run through three concatenated 5 mL HiTrap desalting columns (GE Healthcare, GE17-1408-01) with desalting buffer (50 mM Tris-HCl, pH 7.5, 0.05% Triton X-100) on AKTA protein purification system (GE Healthcare). Protein fractions were collected based on UV280 peak and fractions with high conductivity were discarded.

A final concentration of 150 mM NaCl was added to the desalted extract. The extract was incubated with 100 μL streptavidin magnetic beads (MCE, HY-K0208) on a rotor at 4 °C overnight. The beads were washed with wash buffer (50 mM Tris-HCl, pH 7.5, 150 mM NaCl, 0.5% Triton X-100, 1 mM PMSF) three times and boiled in SDS loading buffer. Four biological replicates were performed for this experiment.

### Mass spectrometry and data analysis

Mass spectrometry was performed as described previously.^20^ Briefly, protein samples were separated by SDS-PAGE and digested in-gel with trypsin (0.5 ng/μL). The peptides were extracted from gel slices, separated by HPLC and sprayed into an LTQ Orbitrap Elite System mass spectrometer (Thermo Fisher Scientific). A database search was performed on the MASCOT server (Matrix Science Ltd) against the IPI (International Protein Index) *Arabidopsis* protein database. The relative amount of each protein was determined by label-free quantification as described.^59^ Briefly, the area of each protein was calculated by averaging the mean area of the three highest peptides from each replicate. The missing values were replaced by the average of minimum values from all replicates.

For proximity labeling data, fold change was calculated by dividing the area of each protein in FUST1-TurboID with the corresponding value in background control YFP-TurboID. The proteins with log_2_Fold-change ≥1 were considered as enriched proteins.

For IP-MS data, fold change was calculated by dividing the area of each protein in FUST1-mVenus with the corresponding value in background control Col-0. *P* value was calculated with the limma package.^60^ Proteins with an adjusted *P* < 0.05 and log_2_Fold-change ≥1 were considered as being enriched by FUST1.

### Co-immunoprecipitation

Approximately 0.5 g of *Nicotiana benthamiana* leaves co-expressing FUST1 with other proteins were ground to a fine powder in liquid nitrogen. The powder was lysed with 2 mL lysis buffer (20 mM Tris-HCl, pH 7.4, 150 mM NaCl, 4 mM MgCl_2_, 0.5% NP-40, 5 mM DTT, 1× Protease Inhibitor Cocktail) at 4 °C for 30 min. After filtration through Miracloth (Millipore, 475855-1RCN) and a brief centrifugation, the supernatant was incubated with 10 μL of FLAG-Nanoab-Magnetic Beads (LABLEAD, FNM-25-1000) for 30 min at 4 °C. The beads were washed four times with lysis buffer and boiled in SDS loading buffer. The immunoprecipitates were subjected to Western blot analyses.

### Western blot analysis

Protein samples were resolved by SDS-PAGE and transferred to PVDF membranes. Antibodies against GFP (Roche, 11814460001) and Flag (Sigma, F1804) were used as primary antibodies. After the primary antibody incubation, horseradish peroxidase (HRP)-conjugated secondary antibodies (GE Healthcare) were used for protein detection by chemiluminescence (Thermo FIsher Scientific, 34095).

### FISH

RNA FISH was performed as previously described.^61^ Roots from seven-day-old seedlings were treated at 37 °C for 1 h, fixed in 4% paraformaldehyde for 30 min at room temperature with gentle vacuum. The roots were washed twice with 1× PBS and arranged onto a slide and covered with a coverslip. The samples were squashed, flash-frozen for ~5 s in liquid nitrogen and air-dried at room temperature for 30 min. The samples were then permeabilized in 70% ethanol for 2 h and washed twice with wash buffer (10% formamide, 2× SSC). 100 µL of hybridization buffer (100 mg/mL dextran sulphate, 10% formamide, 2× SSC) containing 1 µM 5’-Cy5-T_35_ probe was added to each slide and incubated at 45 °C chamber overnight in the dark. After hybridization, the samples were washed twice in wash buffer (10% formamide, 2× SSC) in the dark and imaged by Nikon AX R with NSPARC confocal microscope system using a 100×/1.45 oil objective.

### Reporting summary

Further information on research design is available in the Nature Research Reporting Summary linked to this manuscript.

## Supplementary information

Fig. S1

Fig. S2

Fig. S3

Fig. S4

Fig. S5

Fig. S6

Fig. S7

Fig. S8

Fig. S9

Fig. S10

Fig. S11

Table S1

Table S2

Table S3

Table S4

Supplementary video legends

Video S1

Video S2

## Data Availability

All data are available in the main text or the supplementary materials. The plasmids that support the findings of this study are available from the corresponding author upon reasonable request.
