# Author Correction: A thermosensor FUST1 primes heat-induced stress granule formation via biomolecular condensation in *Arabidopsis*

**DOI:** 10.1038/s41422-025-01134-3

**Published:** 2025-05-29

**Authors:** Pan Geng, Changxuan Li, Xuebo Quan, Jiaxuan Peng, Zhiying Yao, Yunhe Wang, Ming Yang, Yanning Wang, Yunfan Jin, Yan Xiong, Hongtao Liu, Yijun Qi, Peiguo Yang, Kai Huang, Xiaofeng Fang

**Affiliations:** 1 Center for Plant Biology, School of Life Sciences, Tsinghua University, Beijing, China; 2 Institute of Systems and Physical Biology, Shenzhen Bay Laboratory, Shenzhen, Guangdong China; 3 School of Life Sciences, Westlake University, Hangzhou, Zhejiang China; 4 Synthetic Biology Center, Haixia Institute of Science and Technology, Fujian Agriculture and Forestry University, Fuzhou, Fujian China; 5 College of Life Sciences and Oceanography, Shenzhen University, Shenzhen, Guangdong China

**Keywords:** Plant molecular biology, RNA metabolism, Plant signalling

Correction to: *Cell Research* 10.1038/s41422-025-01125-4, published online 14 May 2025

It has come to our attention that in the version of the article initially published, the images for PAB2-labeled SGs in FUST1 WT plants and *fust1-2* mutants in Fig. 5e (left) were mistakenly reused from Fig. 5d (left). This error was due to an inadvertent copy of Fig. 5d for layout templating during figure organization. We have meticulously reviewed our raw data and provided the correct images for Fig. 5e. This correction does not affect the quantification of the results or the conclusions of this study. We sincerely apologize for this oversight and any confusion. The original article has been corrected.


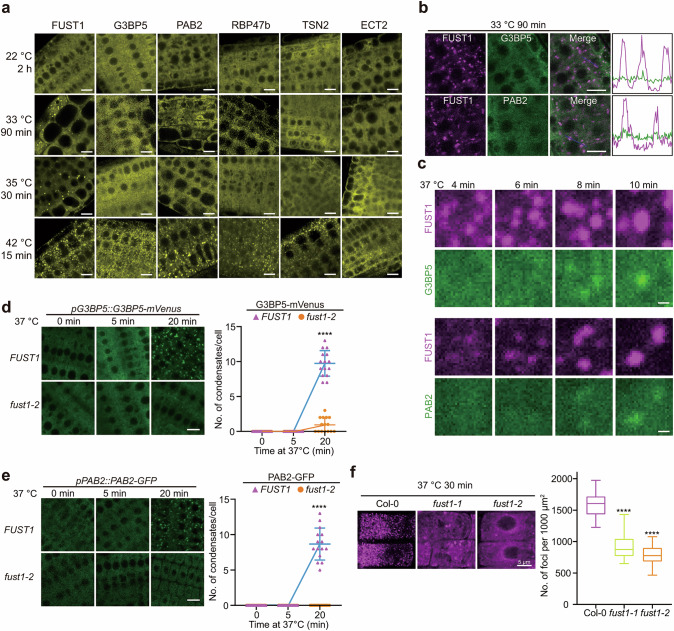


The original article has been corrected.

