# Supplementary material for: A thermosensor FUST1 primes heat-induced stress granule formation via biomolecular condensation in Arabidopsis
Source: Cell Res. 2025 May 14;35(7):483–96. doi: 10.1038/s41422-025-01125-4 (PMC12205081; doi:10.1038/s41422-025-01125-4)
Supplement: Supplementary file 1 — Fig. S1 [file 41422_2025_1125_MOESM1_ESM.pdf]

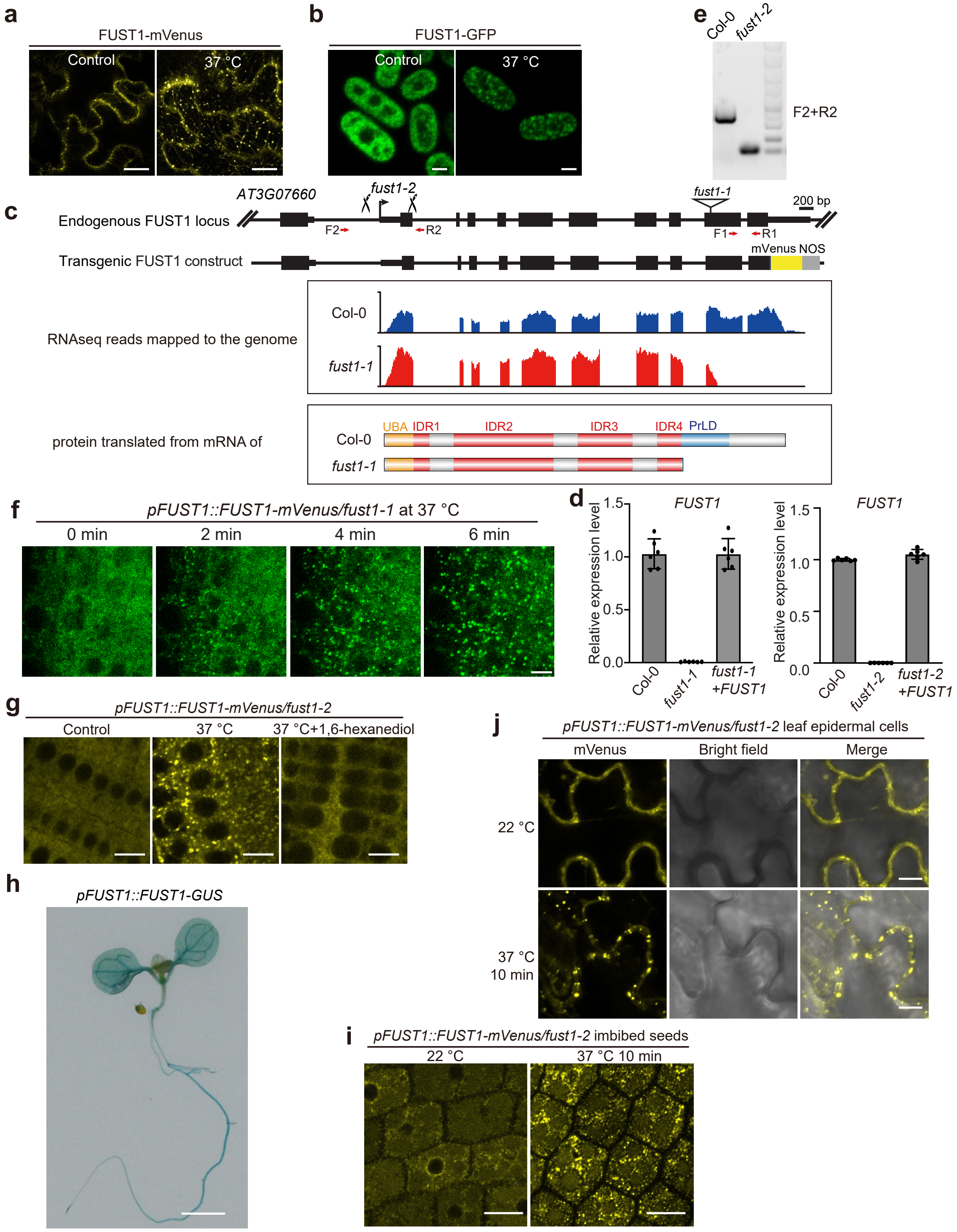

Supplementary Information, Fig. S1 FUST1 undergoes heat-dependent condensation in vivo.
a Representative confocal microscopic images of tobacco epidermal cells expressing FUST1-mVenus. The cells were treated at 37 °C for 10 min. Scale bars, 20 μm. **b** Representative confocal microscopic images of *Schizosaccharomyces pombe* cells expressing FUST1-GFP. The cells were treated at 37 °C for 10 min. Scale bars, 2 μm. **c** Illustration of *FUST1* genomic locus and transgenic *FUST1-mVenus* construct. Thick black boxes indicate exons, thin black boxes indicate UTRs and black lines indicate introns. The position of T-DNA insertion and CRISPR editing are indicated. **d** Relative expression of *FUST1* in the indicated plants. Error bars indicate mean ± SD (n = 6). F1 and R1 indicated in (**c**) were used for amplification. **e** Electrophoresis showing that *fust1-2* has a deletion of 603-bp fragment. F2 and R2 indicated in (**c**) were used for amplification. **f** Time-lapse imaging of FUST1 condensation in *pFUST1::FUST1-mVeuns/fust1-1* root tip cells. Scale bar, 5 μm. **g** Confocal microscopy of *pFUST1::FUST1-mVeuns/fust1-2* root tip cells that are treated as indicated. The seedlings were treated at 37 °C for 5 min to induce the FUST1 condensates, subsequently changed with 10% 1,6-hexanediol at 37 °C for 10 min. Scale bars, 10 μm. **h** The expression pattern of FUST1 as determined by GUS staining of the *pFUST1::FUST1::GUS/Col-0* transgenic plant. Scale bar, 5 mm. **i**, **j** Confocal microscopy of *pFUST1::FUST1-mVeuns/fust1-2* imbibed seeds (**i**) and leaf epidermal cells (**j**) that were treated at 37 °C for 10 min. Scale bars, 10 μm.
