# Supplementary material for: A thermosensor FUST1 primes heat-induced stress granule formation via biomolecular condensation in Arabidopsis
Source: Cell Res. 2025 May 14;35(7):483–96. doi: 10.1038/s41422-025-01125-4 (PMC12205081; doi:10.1038/s41422-025-01125-4)
Supplement: Supplementary file 2 — Fig. S2 [file 41422_2025_1125_MOESM2_ESM.pdf]

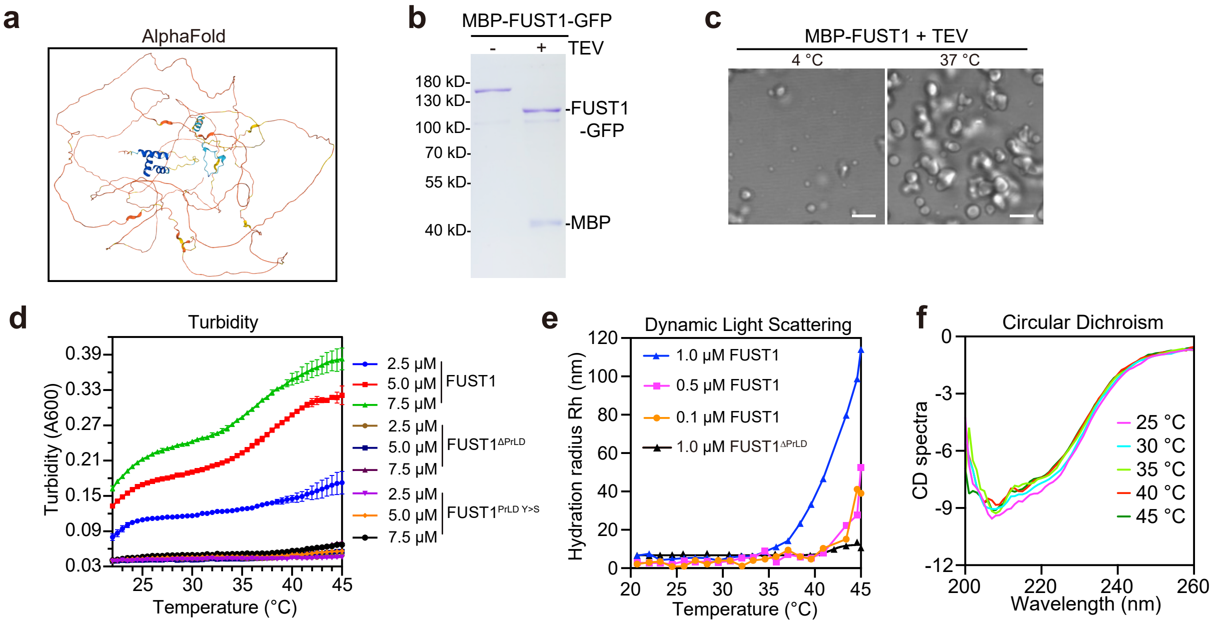

Supplementary Information, Fig. S2 FUST1 undergoes heat-dependent phase separation in vitro.
a The structure of FUST1 as predicted by AlphaFold. **b** Coomassie staining of MBP-FUST1-GFP before and after TEV cleavage to remove the MBP tag. **c** In vitro phase separation assay of 1.0 μM His-FUST1 at 4 °C or 37 °C. Scale bars, 10 μm. **d** Temperature-dependent turbidity analysis of FUST1, FUST1$^{\Delta PrLD}$ and FUST1$^{PrLD\ Y>S}$ with indicated protein concentrations. Error bars indicate mean ± SD (*n* = 3). **e** DLS temperature ramp experiments of FUST1 and FUST1$^{\Delta PrLD}$ with indicated protein concentrations. Turbidity in (**d**) and DLS in (**e**) measurements were conducted in 40 mM Tris-HCl pH 7.4 and 100 mM NaCl. **f** CD spectrum of 0.1 mg/mL (~1.0 μM) His-FUST1 protein at indicated temperatures.
