# Supplementary material for: A thermosensor FUST1 primes heat-induced stress granule formation via biomolecular condensation in Arabidopsis
Source: Cell Res. 2025 May 14;35(7):483–96. doi: 10.1038/s41422-025-01125-4 (PMC12205081; doi:10.1038/s41422-025-01125-4)
Supplement: Supplementary file 3 — Fig. S3 [file 41422_2025_1125_MOESM3_ESM.pdf]

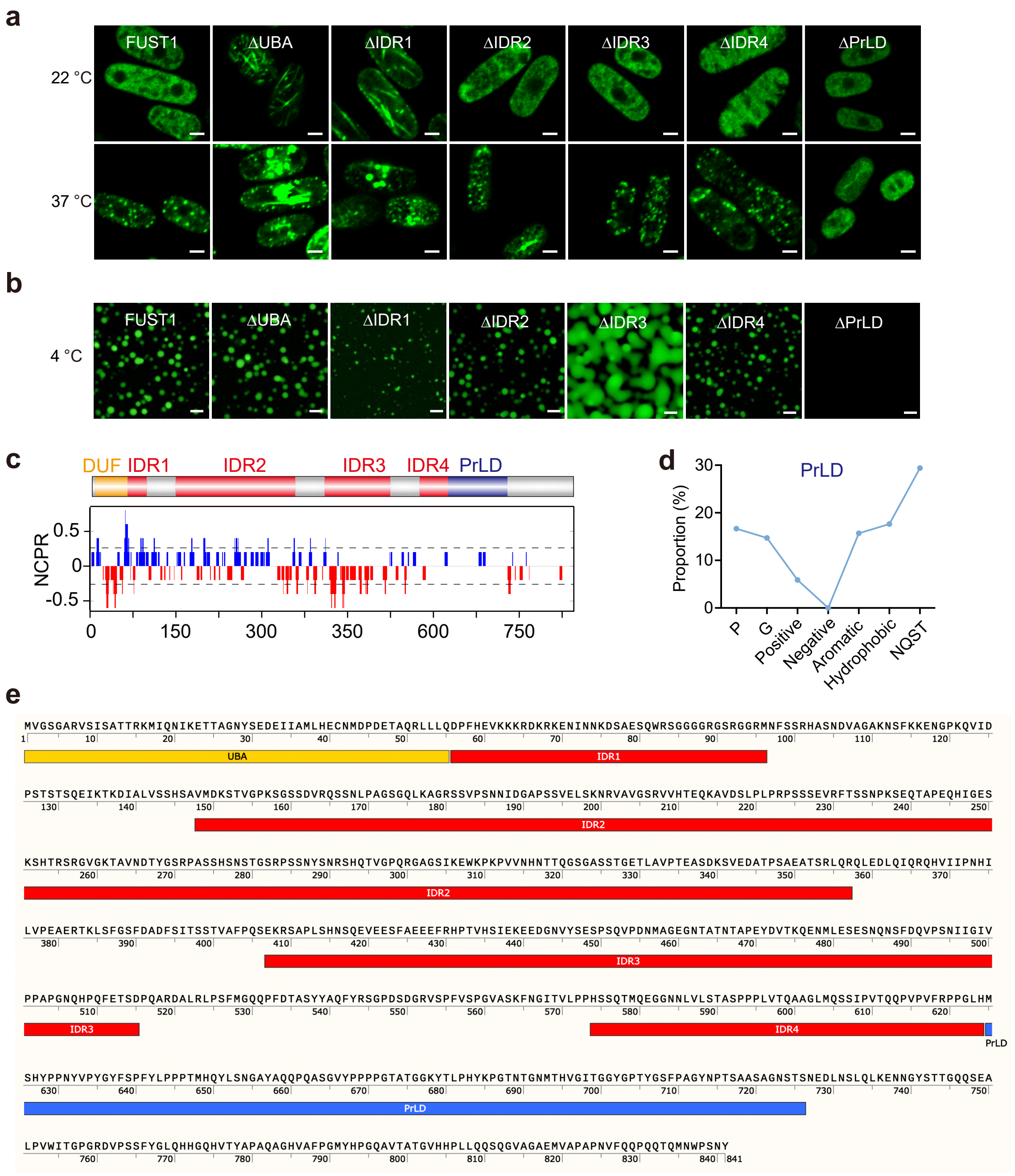

Supplementary Information, Fig. S3 The PrLD is required for FUST1 phase separation.
a Confocal microscopic images of *Schizosaccharomyces pombe* cells expressing FUST1-GFP and its variants. The cells were treated at as indicated. Scale bars, 5 μm. **b** In vitro phase separation of 10.0 μM FUST1-GFP and its variants at 4 °C. Scale bars, 5 μm. **c** Top, the domain structure of FUST1. Bottom, the net charge per residue of FUST1 as analyzed by CIDER website. **d** The amino acid composition of PrLD of FUST1. **e** The amino acid sequence of full-length FUST1. Each domain is indicated.
