# Supplementary material for: A thermosensor FUST1 primes heat-induced stress granule formation via biomolecular condensation in Arabidopsis
Source: Cell Res. 2025 May 14;35(7):483–96. doi: 10.1038/s41422-025-01125-4 (PMC12205081; doi:10.1038/s41422-025-01125-4)
Supplement: Supplementary file 4 — Fig. S4 [file 41422_2025_1125_MOESM4_ESM.pdf]

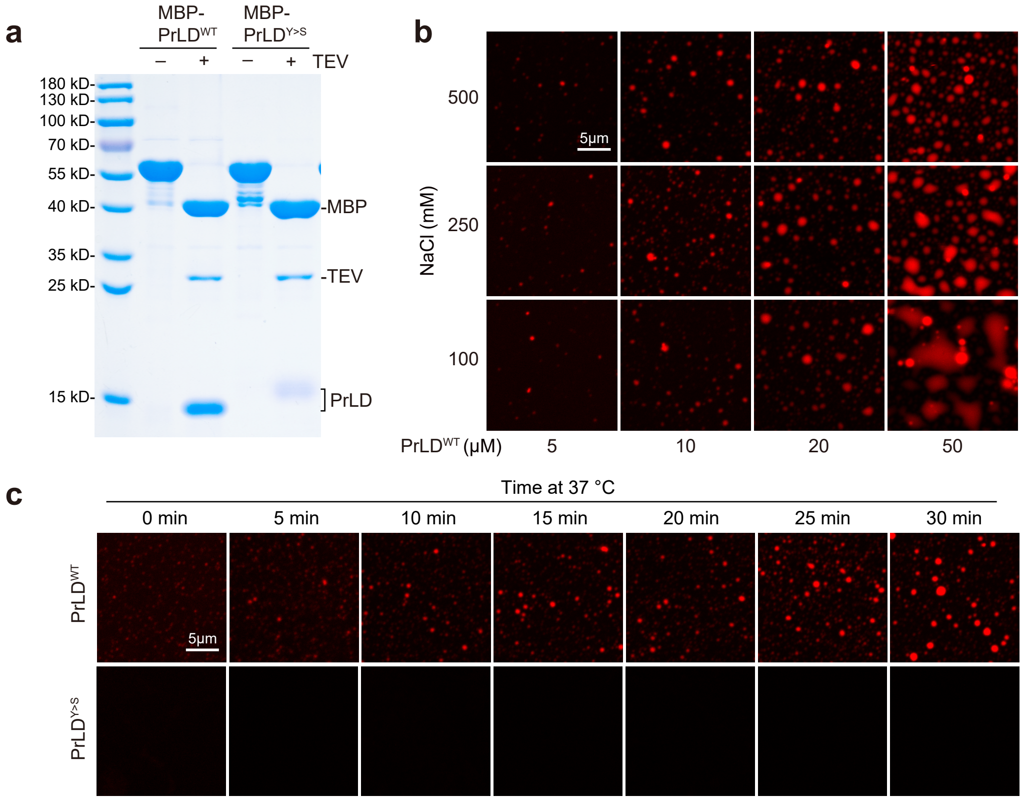

Supplementary Information, Fig. S4 PrLD of FUST1 undergoes heat-dependent condensation in vitro.
a Coomassie staining of purified unlabeled PrLD^{WT} or PrLD$^{Y>S}$ protein. TEV was added to remove the MBP tag. **b** In vitro phase separation assay of Cy5-labelled PrLD^{WT} at indicated protein and salt concentrations. Scale bar, 5 μm. **c** In vitro phase separation assay of 10.0 μM Cy5-labelled PrLD^{WT} or PrLD$^{Y>S}$ at 37 °C. Time points are indicated above. Scale bar, 5 μm.
