# Supplementary material for: A thermosensor FUST1 primes heat-induced stress granule formation via biomolecular condensation in Arabidopsis
Source: Cell Res. 2025 May 14;35(7):483–96. doi: 10.1038/s41422-025-01125-4 (PMC12205081; doi:10.1038/s41422-025-01125-4)
Supplement: Supplementary file 5 — Fig. S5 [file 41422_2025_1125_MOESM5_ESM.pdf]

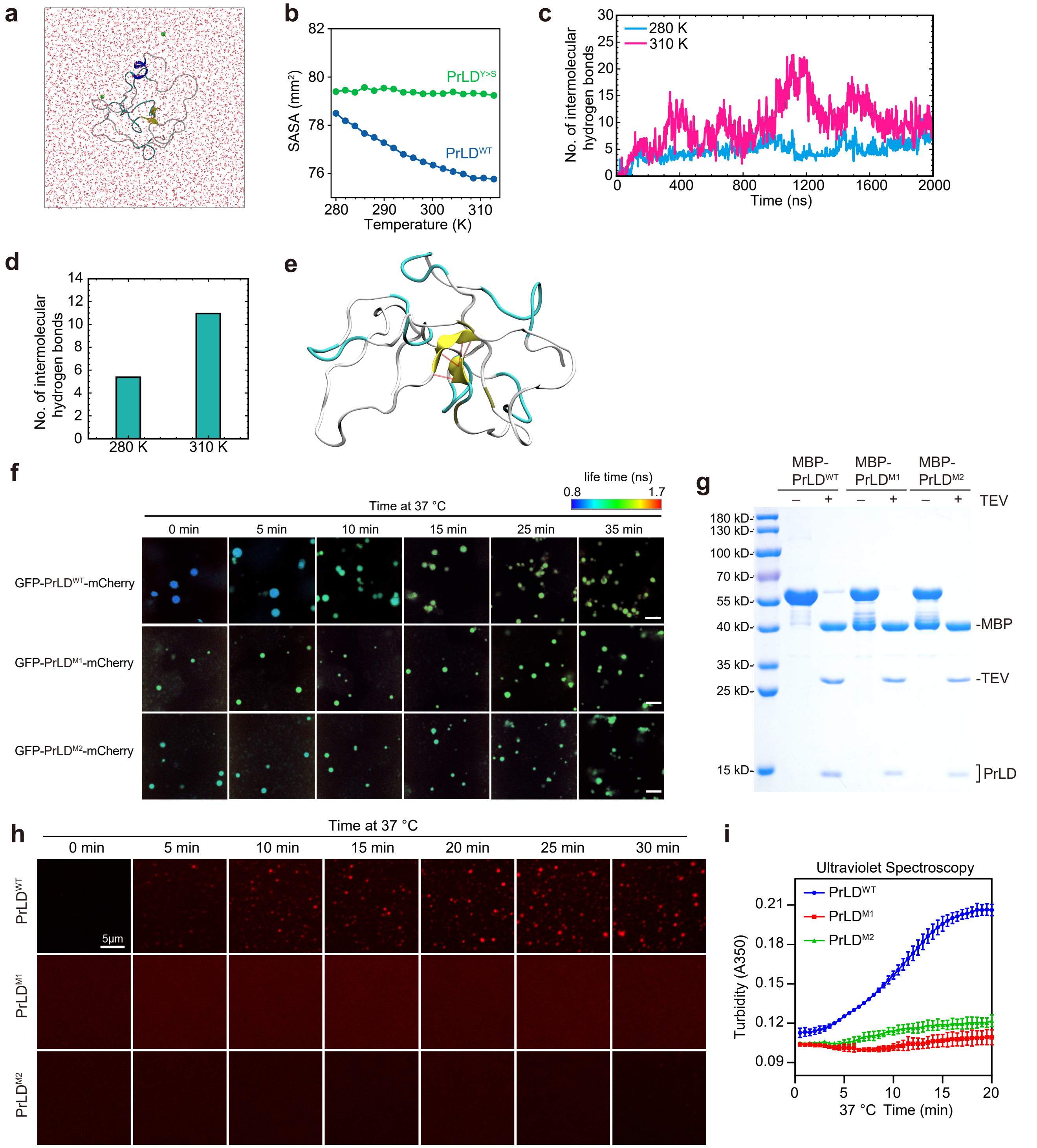

Supplementary Information, Fig. S5 The mechanism of temperature sensing by PrLD.

a Schematic of the MD simulation system. **b** The solvent accessible surface area (SASA) of PrLD^{WT} or PrLD$^{Y>S}$ at elevating temperatures as revealed by MD simulation. **c** The dynamic change of the number of intermolecular hydrogen bonds between two PrLD molecules at 310 K or 280 K as revealed by MD simulation. **d** The average number of intermolecular hydrogen bonds in (**c**). **e** Snapshot of PrLD highlighting the formation of β-strand (yellow). The hydrogen bonds formed between two β-strands were indicated. **f** False-color images of mGFP-life-time of mGFP-PrLD^{WT}-mCherry, mGFP-PrLD^{M1}-mCherry and mGFP-PrLD^{M2}-mCherry at 37 °C in vitro. Scale bars, 3 μm. **g** Coomassie staining of purified unlabeled PrLD^{WT}, PrLD^{M1} and PrLD^{M2}. TEV was added to remove the MBP tag. **h** In vitro phase separation assay of 10.0 μM Cy5-labelled PrLD^{WT}, PrLD^{M1} and PrLD^{M2} at 37 °C. Time points are indicated above. Scale bar, 5 μm. **i** Ultraviolet-visible spectroscopy of 10.0 μM PrLD^{WT}, PrLD^{M1} and PrLD^{M2} at 37 °C. Error bars indicate mean ± SD (*n* = 3).
