# Supplementary material for: A thermosensor FUST1 primes heat-induced stress granule formation via biomolecular condensation in Arabidopsis
Source: Cell Res. 2025 May 14;35(7):483–96. doi: 10.1038/s41422-025-01125-4 (PMC12205081; doi:10.1038/s41422-025-01125-4)
Supplement: Supplementary file 6 — Fig. S6 [file 41422_2025_1125_MOESM6_ESM.pdf]

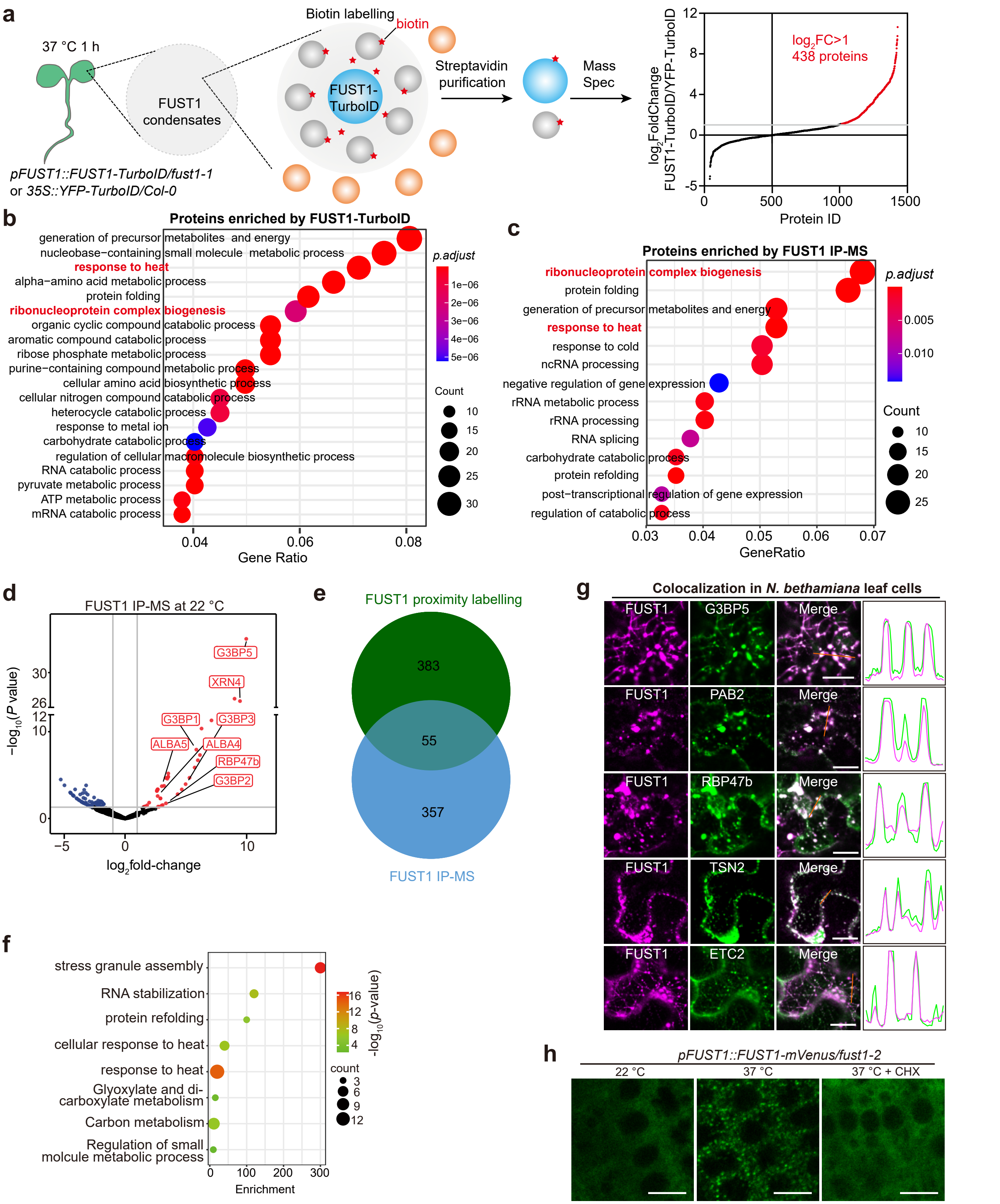

Supplementary Information, Fig. S6 FUST1 interacts with stress granules.

a The procedure of proximity labelling based on Turbo-ID. Both *pFUST1::FUST-TurboID/fust1-1* and *35S::YFP-TurboID/Col-0* plants were treated at 37 °C for 1 h in liquid half-strength MS medium with 50 μM biotin. **b**, **c** Gene Ontology analysis of the proteins significantly enriched by FUST1-TurboID proximity labelling (**b**) and IP-MS (**c**). **d** Volcano plot showing the enrichment of proteins by immunoprecipitation mass spectrometry (IP-MS) of FUST1 in *Arabidopsis* at 22 °C. The *P* values were calculated from 4 biological replicates. **e** Venn diagram showing the overlapped proteins between FUST1 proximity labelling and IP-MS. **f** GO analysis of the overlap proteins in (**e**). **g** Colocalization of indicated proteins with FUST1 in tobacco epidermal cells. Scale bars, 10 μm. **h** Confocal microscopy of *pFUST1::FUST1-mVeuns/fust1-2* root tip cells that are treated as indicated. The seedlings were incubated with 35 μM CHX in liquid half-strength MS medium for 30 min before heat treatment. Scale bars, 10 μm.
