# Supplementary material for: A thermosensor FUST1 primes heat-induced stress granule formation via biomolecular condensation in Arabidopsis
Source: Cell Res. 2025 May 14;35(7):483–96. doi: 10.1038/s41422-025-01125-4 (PMC12205081; doi:10.1038/s41422-025-01125-4)
Supplement: Supplementary file 7 — Fig. S7 [file 41422_2025_1125_MOESM7_ESM.pdf]

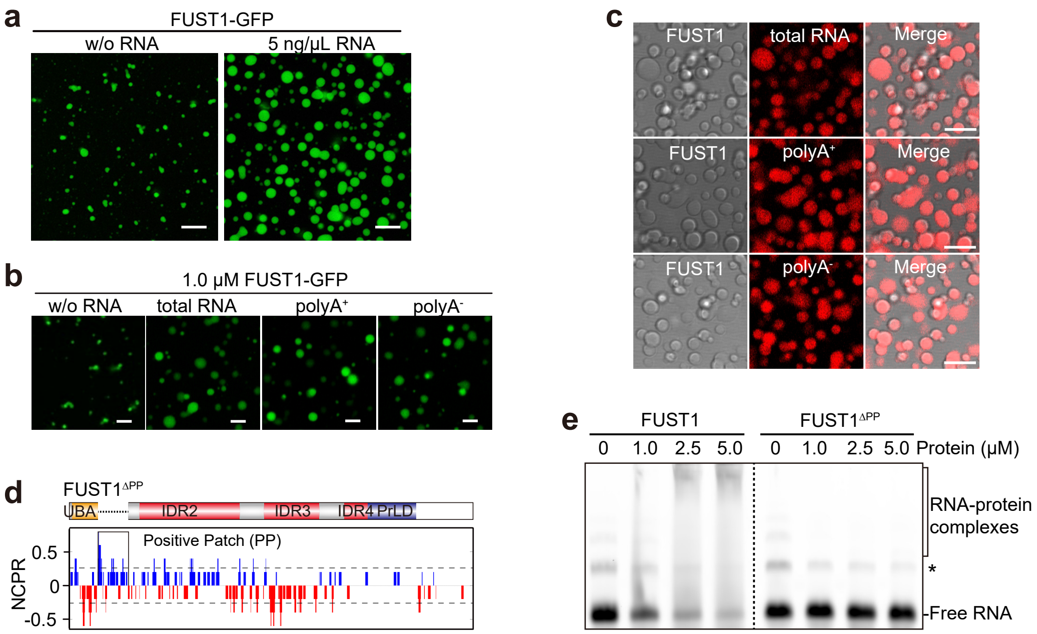

Supplementary Information, Fig. S7 FUST1 condensates partition RNAs.
a In vitro phase separation assay of 2.5 μM FUST1-GFP with or without 5 ng/μL *Arabidopsis* total RNA. Scale bars, 10 μm. **b** In vitro phase separation assay of 2.5 μM FUST1-GFP with or without indicated types of RNA. Scale bars, 5 μm. **c** In vitro phase separation assay showing the partitioning of RNAs by FUST1 droplets. 2.5 μM His-FUST1 and 5 ng/μL RNA were used. **d** Schematic of FUST1-positive patch (pp). **e** EMSA showing the difference binding of FUST1 and FUST1$^{\Delta PP}$ to RNA.
