# Supplementary material for: A thermosensor FUST1 primes heat-induced stress granule formation via biomolecular condensation in Arabidopsis
Source: Cell Res. 2025 May 14;35(7):483–96. doi: 10.1038/s41422-025-01125-4 (PMC12205081; doi:10.1038/s41422-025-01125-4)
Supplement: Supplementary file 8 — Fig. S8 [file 41422_2025_1125_MOESM8_ESM.pdf]

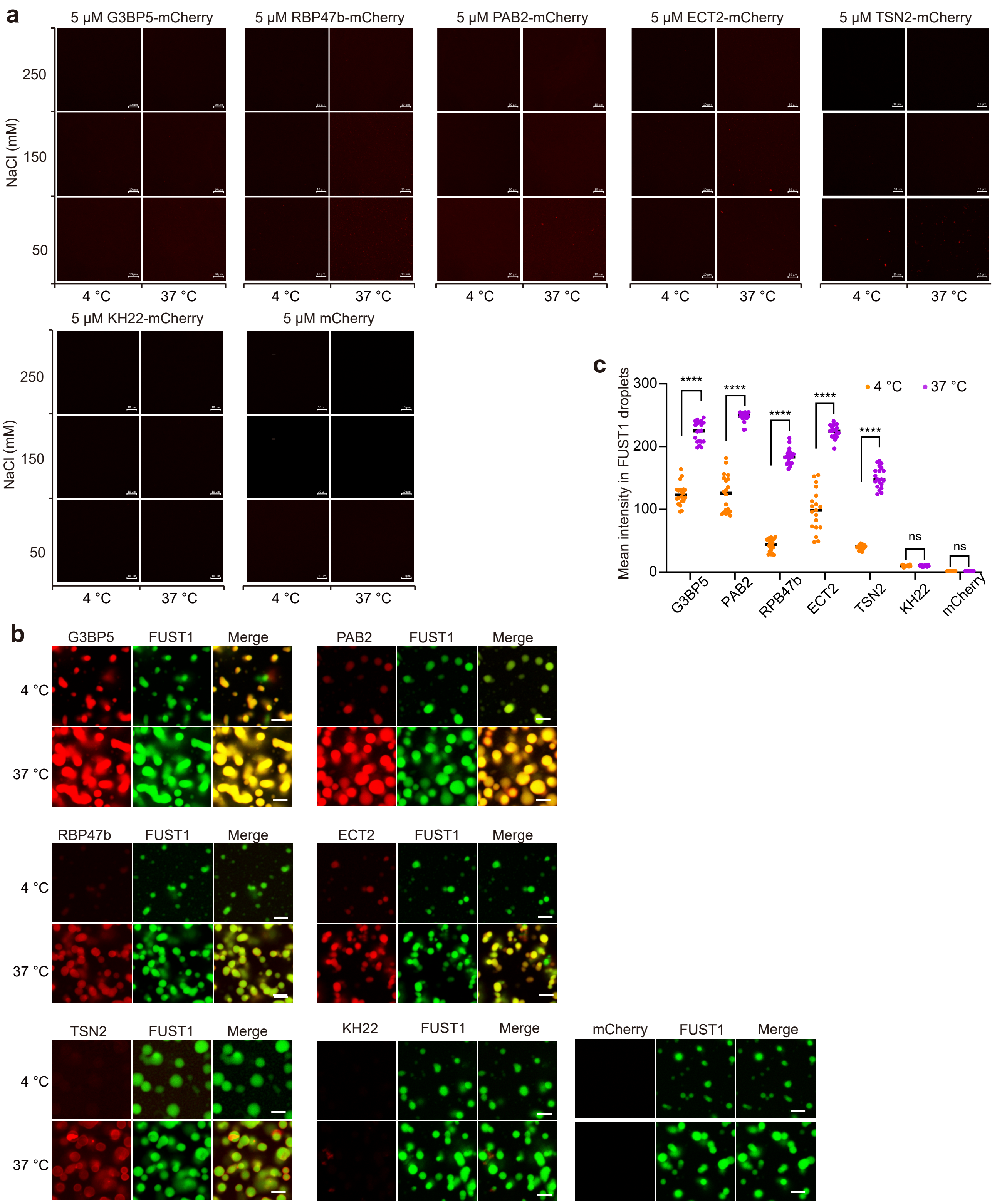

Supplementary Information, Fig. S8 FUST1 condensates partition SG components in vitro.
a Phase diagrams of indicated proteins at different salt concentrations and temperatures. Scale bars, 5 μm. **b** In vitro partitioning of selected proteins by FUST1 droplets after incubation at 4 °C or 37 °C for 30 min. Scale bars, 5 μm. **c** Quantification of the mean intensity of indicated proteins inside FUST1 droplets shown in (**b**) (n = 20). P values were calculated using two-sided Student's t-test. ****$P < 0.0001$.
