# Supplementary material for: A thermosensor FUST1 primes heat-induced stress granule formation via biomolecular condensation in Arabidopsis
Source: Cell Res. 2025 May 14;35(7):483–96. doi: 10.1038/s41422-025-01125-4 (PMC12205081; doi:10.1038/s41422-025-01125-4)
Supplement: Supplementary file 9 — Fig. S9 [file 41422_2025_1125_MOESM9_ESM.pdf]

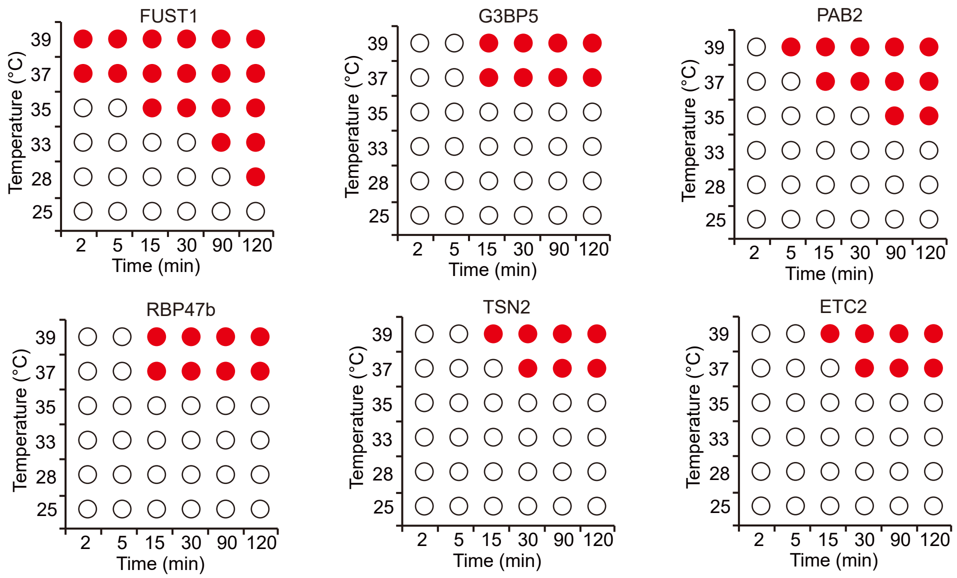


**Supplementary Information, Fig. S9 The kinetics of condensation of FUST1 and SG proteins.**
In vivo condensation diagram of indicated proteins showing the formation of condensates in *Arabidopsis* root tip cells at the indicated temperature and duration of treatment. Red dots, with condensates. Empty circles: without condensates.
