# Supplementary material for: A thermosensor FUST1 primes heat-induced stress granule formation via biomolecular condensation in Arabidopsis
Source: Cell Res. 2025 May 14;35(7):483–96. doi: 10.1038/s41422-025-01125-4 (PMC12205081; doi:10.1038/s41422-025-01125-4)
Supplement: Supplementary file 10 — Fig. S10 [file 41422_2025_1125_MOESM10_ESM.pdf]

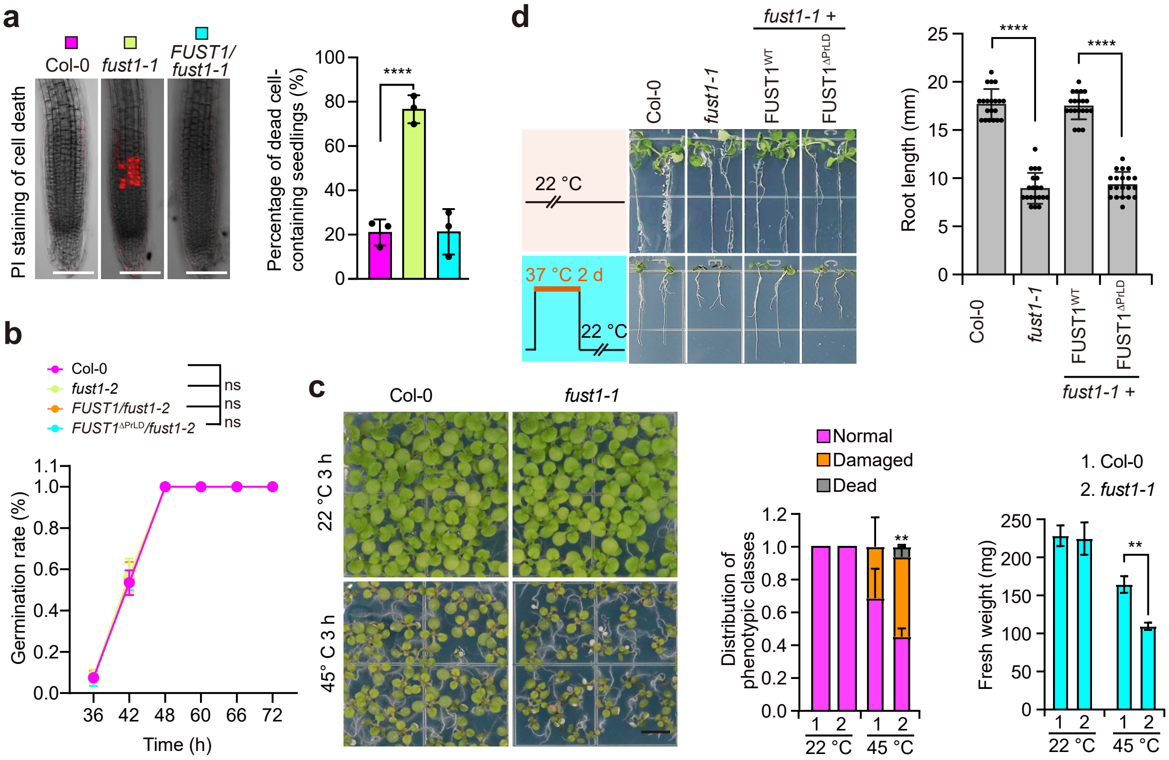

Supplementary Information, Fig. S10 FUST1 is required for basal heat tolerance.

a Left, cell death staining of *Arabidopsis* roots upon heat stress treatment at 42 °C for 2 h. Scale bars, 100 μm. Right, the percentage of seedlings containing cell death staining signal. Error bars indicate mean ± SD (n = 3 independent replicates). Twenty seedlings were assayed in each replicate. P value was calculated using two-sided Student's t-test. ****P < 0.0001. **b** Germination rate of *Arabidopsis* seeds at 22 °C. Error bars indicate mean ± SD (n = 4). Each replicate contains 49 seeds. P values were calculated using two-sided Student's t-test. **c** Left, phenotypes of indicated seedlings assayed for basal heat tolerance. Pictures were taken at seven days of recovery after treatment as indicated. Scale bars, 0.5 cm. Right, quantification of the damage rate and fresh weight of seedlings shown in left. Error bars indicate mean ± SD (n = 3). At least 50 seedlings were assayed for each replicate. P values were calculated using two-sided Student's t-test. **P < 0.01. **d** Left, the root growth phenotype of indicated genotypes after heat stress treatment. Five-day-old seedlings were treated at 37 °C for 2 d and placed at 22 °C for 5 d for recovery. Right, quantification of the root length shown in left. Error bars indicate mean ± SD (n = 20). P values were calculated using two-sided Student's t-test. ****P < 0.0001.
