# Supplementary material for: A thermosensor FUST1 primes heat-induced stress granule formation via biomolecular condensation in Arabidopsis
Source: Cell Res. 2025 May 14;35(7):483–96. doi: 10.1038/s41422-025-01125-4 (PMC12205081; doi:10.1038/s41422-025-01125-4)
Supplement: Supplementary file 11 — Fig. S11 [file 41422_2025_1125_MOESM11_ESM.pdf]

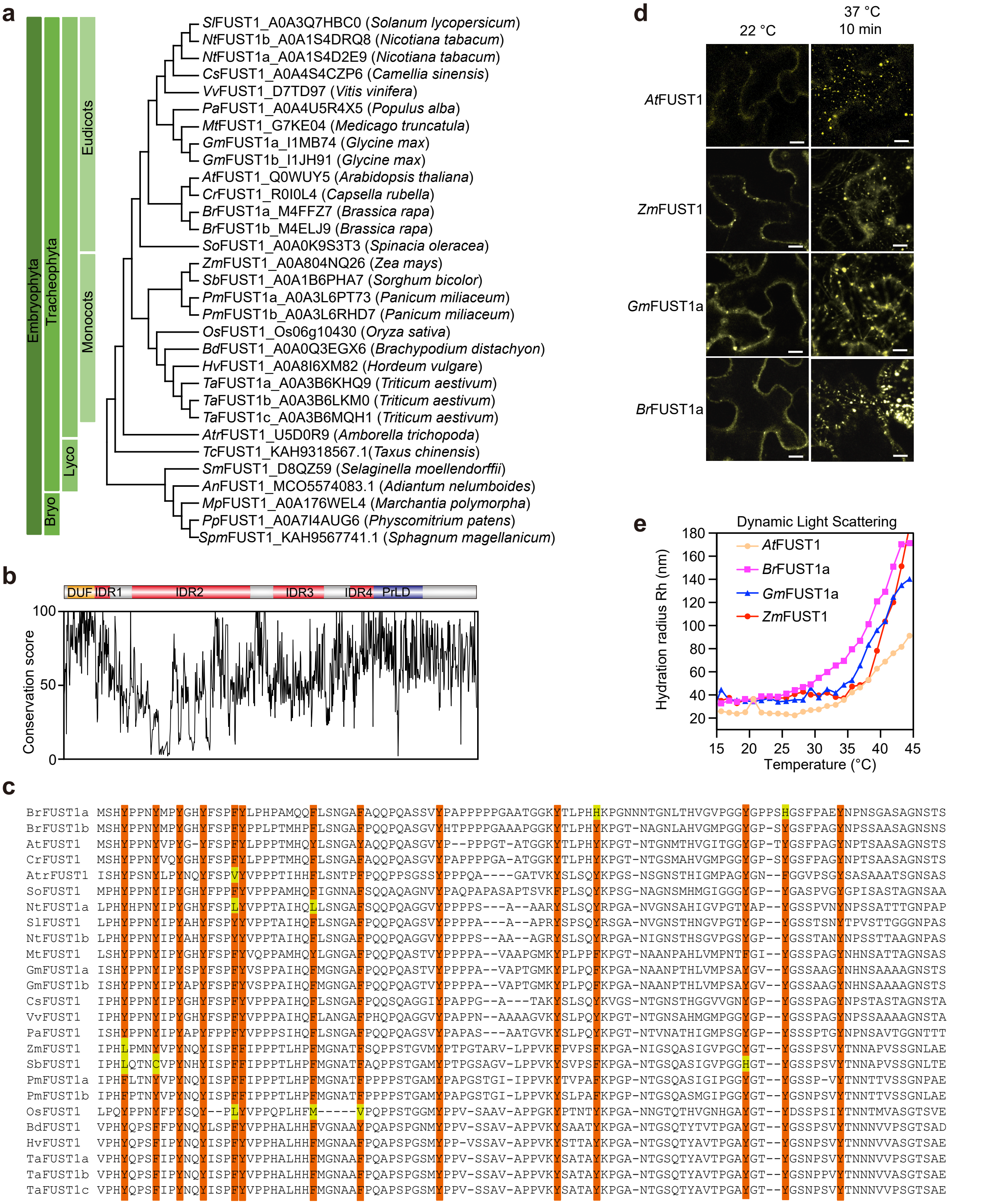

Supplementary Information, Fig. S11 Temperature-dependent condensation of FUST1 is widely conserved.

a The phylogenetic tree of FUST1 homologs inferred using the Neighbor-Joining method. *Arabidopsis* FUST1 was used as query to identify homologues by BLASTp searches against 26 typical Embryophyta genomes on NCBI. Evolutionary analysis was performed in MEGA X. **b** Conservation score for each amino acid between FUST1 homologs. **c** Multiple sequence alignment of the PrLD of FUST1 homologs. **d** Representative confocal microscopic images of tobacco epidermal cells expressing FUST1 homologs. The cells were treated as indicated. Scale bars, 10 μm. **e** DLS temperature ramp experiments of 1.0 μM indicated FUST1 homologs.
