# Supplementary material for: A thermosensor FUST1 primes heat-induced stress granule formation via biomolecular condensation in Arabidopsis
Source: Cell Res. 2025 May 14;35(7):483–96. doi: 10.1038/s41422-025-01125-4 (PMC12205081; doi:10.1038/s41422-025-01125-4)
Supplement: Supplementary file 13 — Table S2 [file 41422_2025_1125_MOESM13_ESM.docx]

**Table S2. SG components identified in FUST1 Turbo-ID**

| Known SG components | | | | | |
| --- | --- | --- | --- | --- | --- |
| Gene ID | Name | Gene ID | Name | Gene ID | Name |
| AT3G07660 | FUST1 | AT1G69250 | G3BP4 | AT3G13460 | ECT2 |
| AT1G26110 | DCP5 | AT1G13730 | G3BP5 | AT5G61020 | ECT3 |
| AT3G13300 | VCS | AT2G03640 | G3BP6 | AT3G17330 | ECT6 |
| AT1G79090 | PAT1 | AT5G48650 | G3BP7 | AT1G48110 | ECT7 |
| AT3G58570 | RH52 | AT4G34110 | PAB2 | AT1G79270 | ETC8 |
| AT1G02080 | NOT1 | AT2G23350 | PAB4 | AT5G07350 | TSN1 |
| AT5G18230 | NOT3 | AT1G49760 | PAB8 | AT5G61780 | TSN2 |
| AT5G12980 | NOT9B | AT1G54080 | UBP1A | AT1G20220 | ALBA4 |
| AT5G47010 | UPF1 | AT1G17370 | UBP1B | AT1G76010 | ALBA5 |
| AT5G21160 | LARP1A | AT3G14100 | UBP1C | AT1G54170 | CID3 |
| AT2G45620 | URT1 | AT1G49600 | RBP47A | AT3G14010 | CID4 |
| AT1G24050 | K7EMZ9 | AT3G19130 | RBP47B | AT2G26280 | CID7 |
| AT5G60980 | G3BP1 | AT5G54900 | RBP45A | AT4G36020 | CSP1 |
| AT5G43960 | G3BP2 | AT4G27000 | RBP45C | AT2G17870 | CSP3 |
| AT3G25150 | G3BP3 | AT5G19350 | RBP45D | AT5G64960 | CDKC2 |
| Translation factors | | Translation-related | | Chaperons | |
| Gene ID | Name | Gene ID | Name | Gene ID | Name |
| AT4G01290 | CBE1 | AT2G40510 | RPS26B | AT1G07400 | HSP17.8 |
| AT5G42950 | MUSE11 | AT5G09510 | RPS15D | AT1G16030 | HSP70-5 |
| AT3G60240 | EIF4G | AT1G15250 | RPL37A | AT1G74310 | HSP101 |
| AT3G11400 | EIF3G1 | AT2G19730 | RPL28A | AT3G12580 | HSP70-4 |
| AT4G20980 | EIF3-7 | AT5G02610 | RPL35D | AT2G29500 | HSP17.6B |
| AT1G09640 | EF1G1 | AT2G40590 | RPS26A | AT5G56030 | HSP90-2 |
| AT5G19510 | EF1B2 | AT3G09500 | RPL35A | AT3G46230 | HSP17.4 |
| AT1G30230 | EF1Bb | AT4G29410 | RPL28C | AT2G04030 | HSP90.5 |
| AT5G18110 | NCBP | AT2G39390 | RPL35B | AT4G24280 | HSP70-6 |
| AT5G20920 | EIF2B | AT3G09200 | RPP0B | AT3G09440 | HSP70-3 |
| AT5G57870 | EIFiso4G1 | AT3G09200 | RPP0B | AT3G23990 | HSP60 |
| AT2G39990 | EIF2F | AT3G44890 | RPL9 | AT3G20050 | CCT1 |
| AT1G76810 | EIF5B1 | AT4G28360 | UL22MY | AT4G09150 | CCT11 |
| AT3G13920 | EIF4A-1 | AT4G17300 | ATNS1 | AT3G18190 | CCT4 |
| AT1G12920 | ERF1-2 | AT5G56680 | SYNC1 | AT1G24510 | CCT5 |
| AT3G59410 | GCN2 | AT4G26300 | tRNA ligase | AT5G16070 | CCT6-1 |
| AT2G40290 | EIF2A | AT3G46100 | tRNA ligase | AT3G02530 | CCT6-2 |
| AT5G27640 | EIF3B | AT4G33760 | OKI | AT3G11830 | CCT7 |
|  |  | AT1G50200 | tRNA ligase | AT3G03960 | CCT8 |
|  |  | AT1G09620 | tRNA ligase | AT5G20890 | CCT2 |
|  |  | AT1G29880 | tRNA ligase | AT2G28000 | CPN60A |
|  |  |  |  | AT1G55490 | CPN60B |
