# Supplementary material for: A thermosensor FUST1 primes heat-induced stress granule formation via biomolecular condensation in Arabidopsis
Source: Cell Res. 2025 May 14;35(7):483–96. doi: 10.1038/s41422-025-01125-4 (PMC12205081; doi:10.1038/s41422-025-01125-4)
Supplement: Supplementary file 15 — Table S4 [file 41422_2025_1125_MOESM15_ESM.docx]

**Table S4. Primers and oligos used in this work.**

| Primer Name | Sequence (5’-3’) | Purpose |
| --- | --- | --- |
| F1 | GGCTACAGTACAACAGGGCA | Quantitative RT-PCR analysis |
| R1 | GCGGATGATGGACTCCTGTT |  |
| Tubulin-F | ATCCGTGAAGAGTACCCAGAT | Quantitative RT-PCR analysis |
| Tubulin-R | AAGAACCATGCACTCATCAGC |  |
| F2 | GGGTAACATCGTTTGAGATAG | Genotyping of *fust1-2* |
| R2 | gattatacCCTGAAGAAGTAAGCG |  |
| F3 | CCGCATTCATCTCAAACCATGC | Genotyping of *fust1-1* |
| R3 | CAGTAATTACTTGGCCAAT |  |
| FUST1-sgRNA-F1 | GATTGACTGCTGGGAACTATAGCG | *pAtU6-FUST1-1* |
| FUST1-sgRNA-R1 | AAACGCGCTATAGTTCCCAGCAGT |  |
| FUST1-sgRNA-F2 | GATTGGCTCAACGCTTACTTCTTC | *pAtU6-FUST1-2* |
| FUST1-sgRNA-R2 | AAACGGAAGAAGTAAGCGTTGAGC |  |
| pFUST1-GUS-F | GACCATGATTACGCCAAGCTTCAGTATAAAAGCG | *pFUST1-GUS* |
| pFUST1-GUS-R | AAAACGACGGCCAGTCCTCTCTTCTATCAAACG |  |
| pFUST1-F | ACGACGGCCAGTGCCAAGCTTCAGTATAAAAGCGAGTAAGGTTG | *pFUST1:gFUST1-mVenus* |
| FUST1-R | GGATGCGGCAGCAGAAAGCTTGTAATTACTTGGCCAATTCATCTG |  |
| pFUST1-F2 | tacgtttgatagaagagaggATGGTCGGCAGTGGCGCTAG | *pFUST1:gFUST1-TurboID* |
| FUST1-R2 | gcgcccacccttagcGTAATTACTTGGCCAATTCATCTG |  |
| pFUST1-F | ACGACGGCCAGTGCCAAGCTTCAGTATAAAAGCGAGTAAGGTTG | *proFUST1:gFUST1ΔPrLD-mVenus* |
| FUST1ΔPrLD-R | GGATGCGGCAGCAGAAAGCTTGTAATTACTTGGCCAATTCATCTG |  |
| FUST1-CDS-F | ATCTATCTCTCTCGAGGTACCATGGTCGGCAGTGGCGCT | *35S:FUST1-mVenus* |
| FUST1-CDS-R | TGCCGCGGATGCGGCAGCAGAGTAATTACTTGGCCAATTCATCTGTG |  |
| FUST1-ΔDUF-F | ATCTATCTCTCTCGAATGGTCGGCAGTGGCGCTAGGGTTTCGAACATCAACAACAAGG | *35S:FUST1ΔDUF-mVenus* |
| FUST1-CDS-R | TGCCGCGGATGCGGCAGCAGAGTAATTACTTGGCCAATTCATCTGTG |  |
| FUST1-ΔIDR1-F | CATGAGGTTGAGATAAAAACGAAAGATATAG | *35S:FUST1ΔIDR1-mVenus* |
| FUST1-ΔIDR1-R | GTTTTTATCTCAACCTCATGGAATGGATC |  |
| FUST1-ΔIDR2-F | CCATTCTGCTCAGCTTGAAGACTTGCAG | *35S:FUST1ΔIDR2-mVenus* |
| FUST1-ΔIDR2-R | CTTCAAGCTGAGCAGAATGGCTTGAG |  |
| FUST1-ΔIDR3-F | TCCTCAGAGTTCTGACCCTCAAGCACG | *35S:FUST1ΔIDR3-mVenus* |
| FUST1-ΔIDR3-R | GAGGGTCAGAACTCTGAGGAAAGGCTAC |  |
| FUST1-ΔIDR4-F | ATTGCCTCCGATGTCGCATTATCCACC | *35S:FUST1ΔIDR4-mVenus* |
| FUST1-ΔIDR4-R | AATGCGACATCGGAGGCAATACTGTAATC |  |
| FUST1-CDS-F | ATCTATCTCTCTCGAGGTACCATGGTCGGCAGTGGCGCT | *35S:FUST1ΔPrLD-mVenus* |
| FUST1-ΔPrLD-R | GGATGCGGCAGCAGAGGATGCGGCAGCAGAAAGC |  |
| pG3BP5-F | ACGACGGCCAGTGCCGGAACTCTGAACTCTGACAG | *pG3BP5:gG3BP5-mVenus* |
| G3BP5-R | GCGCCCACCCTTAGCACGGCTAGCTTCTACCG |  |
| pRBP47B-F | ACGACGGCCAGTGCCGAGCAGGTTTCTTCTGTGAA | *pRBP47B:gRBP47B-mVenus* |
| RBP47B-R | GGATGCGGCAGCAGACCTCGAGGAGAGTCTCTG |  |
| pECT2-F | acgacggccagtgccAAGCTTAACCTATATTTCCAATGACATAAC | *pECT2:gECT2-mVenus* |
| ECT2-R | TCCTCCCTGCAGCGCGGTACCGCAACCATTTGCCACCACATC |  |
| pTSN2-F | acgacggccagtgccAAGCTTAACAACTCAACCCATAAAATTAAA | *pTNS2:gTSN2-mVenus* |
| TSN2-R | TCCTCCCTGCAGCGCGGTACCcccgcgacccggtttcctgac |  |
| FUST1-pnmt-F | GTCGCTTTGTTAAAGCTAGCATGGTCGGCAGTGGCG | *pmnt1:FUST1(variants)-yeGFP* |
| FUST1-yeGFP-R | TCACCTTTAGACATGGATCCGTAATTACTTGGCCAATTC |  |
| G3BP1-F | ATCTATCTCTCTCGAATGGCACAGCAGGAAGC | *35S:G3BP1-mVenus* |
| G3BP1-R | GGATGCGGCAGCAGAAGATGAACCACCACCTCG |  |
| G3BP2-F | ATCTATCTCTCTCGAATGGCGACTCCTTATCCTG | *35S:G3BP2-mVenus* |
| G3BP2-R | GGATGCGGCAGCAGAGCGACCACCACCGCGGTAG |  |
| G3BP4-F | ATCTATCTCTCTCGAATGGCTACCGAGGGAGTTG | *35S:G3BP4-mVenus* |
| G3BP4-R | GGATGCGGCAGCAGAATGTGCGGCTTCACTTTTTTTC |  |
| G3BP5-F | ATCTATCTCTCTCGAATGGCACTTGAATCAAATG | *35S:G3BP5-mVenus* |
| G3BP5-R | GGATGCGGCAGCAGAACGGCTAGCTTCTACCG |  |
| G3BP6-F | ATCTATCTCTCTCGAATGACACCTGAATCAAACG | *35S:G3BP6-mVenus* |
| G3BP6-R | GGATGCGGCAGCAGAGTTTTTGGCCTCAGTATTGC |  |
| PAB2-F | TCTCTCTCGAGGTACCATGGCGCAGGTTCAACTTC | *35S:PAB2-mVenus* |
| PAB2-R | CGCCCACCCTTAGCACTAGTAGAGAGGTTCAAGGAAGC |  |
| PAB4-F | ATCTATCTCTCTCGAATGGCTCAGGTTCAAGCTC | *35S:PAB4-mVenus* |
| PAB4-R | GGATGCGGCAGCAGATAAATGATCATTGATGGAAAGTG |  |
| PAB8-F | ATCTATCTCTCTCGAATGGCTCAGATTCAGCATC | *35S:PAB8-mVenus* |
| PAB8-R | GGATGCGGCAGCAGAAGGTACGATGTTGTCTCC |  |
| RBP47b-F | TCTCTCTCGAGGTACCATGCAGACAACCAACGGC | *35S:RBP47B-mVenus* |
| RBP47b-R | CGCCCACCCTTAGCACTAGTATTCTCCCCATGATAGTTGT |  |
| ECT2-F | TCTCTCTCGAGGTACCATGGCTACCGTTGCTCCTC | 35S:ECT2-mVenus |
| ECT2-R | gcccacccttagcACTAGTGCAACCATTTGCCACCAC |  |
| TSN2-F | TCTCTCTCGAGGTACCATGGCGACTGGGGCAGC | 35S:TSN2-mVenus |
| TSN2-R | cgcccacccttagcACTAGTCCCGCGACCCGGTTTCC |  |
| FUST1-TEV-F | CTCTACTTCCAATCCGCTAGCATGGTCGGCAGTGGCG | MBP-HIS-FUST1-GFP |
| FUST1-EGFP-R | TTGCTCACTTTCATGGATCCGTAATTACTTGGCCAATTC |  |
| FUST1-HIS-F | GCGGCAGCCATATGGCTAGCATGGTCGGCAGTGGCGC | HIS-FUST1 |
| FUST1-HIS-R | GTGGTGGTGGTGGTGGTAATTACTTGGCCAATTC |  |
| FUST1-HIS-F | GCGGCAGCCATATGGCTAGCATGGTCGGCAGTGGCGC | MBP-HIS-FUST1 |
| FUST1-HIS-R | GTGGTGGTGGTGGTGGTAATTACTTGGCCAATTC |  |
| FUST1-HIS-F | GCGGCAGCCATATGGCTAGCATGGTCGGCAGTGGCGC | MBP-HIS-FUST1ΔPrLD |
| FUST1-HIS-R | GTGGTGGTGGTGGTGGTAATTACTTGGCCAATTC |  |
| G3BP5-mC-F | CAAATGGGTCGCGGATCCATGGCACTTGAATCAAATG | MBP-His-G3BP5-mCherry |
| G3BP5-mC-R | GCCCTTGCTCACTTTCATACGGCTAGCTTCTACCG |  |
| PAB2-mC-F | CAAATGGGTCGCGGATCCATGGCGCAGGTTCAAC | MBP-His-PAB2-mCherry |
| PAB2-mC-R | GCCCTTGCTCACTTTCATAGAGAGGTTCAAGGAAGC |  |
| RBP47b-mC-F | CAAATGGGTCGCGGATCCATGCAGACAACCAACGGC | MBP-His-RBP47b-mCherry |
| RBP47b-mC-R | GCCCTTGCTCACTTTCATATTCTCCCCATGATAGTTG |  |
| ECT2-mC-F | CAAATGGGTCGCGGATCCATGGCTACCGTTGCTCC | MBP-His-ECT2-mCherry |
| ECT2-mC-R | GCCCTTGCTCACTTTCATGCAACCATTTGCCACCAC |  |
| TSN2-mC-F | CAAATGGGTCGCGGATCCATGGCGACTGGGGCAG | MBP-His-TSN2-mCherry |
| TSN2-mC-R | GCCCTTGCTCACTTTCATCCCGCGACCCGGTTTC |  |
| mGFP-FUST1-PrLD^WT^-mC-F | GAAAGGCGGTAGCGGTGGATCCATGTCGCATTATCCACC | MBP-His-mGFP-FUST1-PrLD^WT^-mCherry-His |
| mGFP-FUST1-PrLD^WT^-mC-R | ATGTTATCCTCCTCGCCCTTGCTCACTTTCATGGATCC GGAAGTCGAATTCCCGGC |  |
| mGFP-FUST1-PrLD^YS^-mC-F | GAAAGGCGGTAGCGGTGGATCCATGTCGCATTCGCCACC | MBP-His-mGFP-FUST1-PrLD^YS^-mCherry-His |
| mGFP-FUST1-PrLD^YS^-mC-R | ATGTTATCCTCCTCGCCCTTGCTCACTTTCATGGATCC GGAAGTCGAATTCCCGGC |  |
| mGFP-FUST1-PrLD^WT^-F | GAAAGGCGGTAGCGGTGGATCCATGTCGCATTATCCACC | MBP-His-mGFP-FUST1-PrLD^WT^-His |
| mGFP-FUST1-PrLD^WT^-R | GTGGTGGTGGTGCTCGAGGGAAGTCGAATTCCCGGC |  |
| mGFP-FUST1-PrLD^YS^-F | GAAAGGCGGTAGCGGTGGATCCATGTCGCATTCGCCACC | MBP-His-mGFP-FUST1-PrLD^YS^-His |
| mGFP-FUST1-PrLD^YS^-R | GTGGTGGTGGTGCTCGAGGGAAGTCGAATTCCCGGC |  |
| FUST1-PrLD^WT^-F | GCGGCAGCCATATGGCTAGCATGTCGCATTATCCACC | MBP-FUST1-PrLD^WT^-His |
| FUST1-PrLD^WT^-R | TGGTGGTGGTGGTGCTCGAGGGAAGTCGAATTCCCGG |  |
| FUST1-PrLD^YS^-F | GCGGCAGCCATATGGCTAGCATGTCGCATTCGCCACC | MBP-FUST1-PrLD^YS^-His |
| FUST1-PrLD^YS^-R | TGGTGGTGGTGGTGCTCGAGGGAAGTCGAATTCCCGG |  |
| UBAP2L-PrLD-F | CTCTACTTCCAATCCGCTAGCATGGCCCAACCCCAACAG | MBP-UBAP2L-PrLD-His |
| UBAP2L-PrLD-R | GTGGTGGTGGTGGTGCTCGAGGTTGGCCCCCCAGCTGTAG |  |
| UBAP2L-F | CGAGCTCAAGCTTCGGAATTCATGATGACATCGGTGGGC | PCDH-CMV-UBAP2L∆PrLD -mCherry |
| UBAP2L-R | CAGAGGTTGATTGGATCCCTAGTTGGCCCCCCAGCTGTA |  |
| UBAP2L∆PrLD-F | GGGGTCCCGCCGTTGTTGCCTTAGGGATCCAATCAACCT | PCDH-CMV-UBAP2L∆PrLD -mCherry |
| UBAP2L∆PrLD-R | AGGCAACAACGGCGGGACCCCAGGAGGGAGGTTGGGAGG |  |
