# Supplementary material for: A thermosensor FUST1 primes heat-induced stress granule formation via biomolecular condensation in Arabidopsis
Source: Cell Res. 2025 May 14;35(7):483–96. doi: 10.1038/s41422-025-01125-4 (PMC12205081; doi:10.1038/s41422-025-01125-4)
Supplement: Supplementary file 16 — Supplementary video legends [file 41422_2025_1125_MOESM16_ESM.docx]

**Video S1. Time lapse imaging of FUST1 condensation in *Arabidopsis*.**

Five-day-old *pFUST1::FUST1-mVenus/fust1-1* vertically-grown seedlings were imaged in a chamber with temperature at 37°C.

**Video S2. FRAP of FUST1 condensates in *Arabidopsis* root tip cells.**

Five-day-old *pFUST1::FUST1-mVenus/fust1-1* vertically-grown seedlings were treated at 37°C for 10 min before being imaged.
